# Diet, gut microbiota, and the gut-brain axis: mechanistic interactions and therapeutic implications in neuropsychiatric disorders

**DOI:** 10.3389/fcimb.2026.1834069

**Published:** 2026-06-19

**Authors:** Xin Wang, Yinzi Piao, Baoguo Xia, Weixin Chu, Xiaohua Yao, Weisheng Li

**Affiliations:** 1 Department of Pediatrics, Qingdao Municipal Hospital, University of Health and Rehabilitation Sciences, Qingdao, China; 2 Department of Otolaryngology-Head and Neck Surgery, Qingdao Municipal Hospital, University of Health and Rehabilitation Sciences, Qingdao, China; 3 Department of Gynecology, Qingdao Municipal Hospital, University of Health and Rehabilitation Sciences, Qingdao, China

**Keywords:** dysbiosis, microbial metabolites, microbiota-gut-brain axis, neuroinflammation, neuropsychiatric disorders, precision nutrition

## Abstract

The gut microbiota is a dynamic trans-kingdom ecosystem that contributes to host immunological, metabolic, and neuroendocrine homeostasis through the microbiota-gut-brain axis (MGBA). Diet is one of the major environmental factors shaping this axis, as it influences microbial composition, microbial production of neuroactive metabolites, and intestinal barrier integrity. Dysbiosis has been increasingly associated with neurological, psychiatric, and neurodevelopmental disorders, including Alzheimer’s disease, Parkinson’s disease, depression, autism spectrum disorder, and attention-deficit/hyperactivity disorder. Experimental studies have identified several potential mechanisms linking gut microbiota to brain function, including immune modulation, vagus nerve signaling, microbial metabolite production, and blood-brain barrier regulation. However, translating these findings into clinical practice remains challenging because human studies are affected by genetic heterogeneity, dietary variation, medication use, lifestyle factors, and disease-specific confounders. In this review, we summarize current evidence on the interactions among diet, gut microbiota, and brain function, with particular emphasis on microbial metabolites, immune mediators, and barrier-related mechanisms. We also critically discuss microbiota-targeted interventions, including precision nutrition, probiotics, and fecal microbiota transplantation, highlighting both their therapeutic potential and their current limitations. A more cautious and mechanistically integrated understanding of the MGBA may support the development of personalized strategies for neuropsychiatric disease prevention and management.

## 1 Introduction

Humans and other animals are colonized by diverse microbial communities, including bacteria, fungi, and viruses, with the gastrointestinal tract representing one of the most densely colonized microbial habitats. Accumulating evidence indicates that the gut microbiota is not merely a passive commensal community but a dynamic regulator of organ development, immune maturation, and metabolic homeostasis. The composition and function of this microbial ecosystem are continuously shaped by host age, diet, medication exposure, and other environmental factors (Wastyk et al., 2021; Ronan et al., 2021; Cai et al., 2022).

The gut microbiota communicates with the brain through multiple interconnected routes, including neural, endocrine, immune, and metabolic signaling. Neural communication is mediated in part by the autonomic nervous system and the enteric nervous system (ENS), which integrate visceral sensory information and coordinate local digestive, endocrine, and immune functions (Bruning et al., 2020; Nguyen et al., 2023). In parallel, microbial and host-derived metabolites can influence neuroactive signaling. The gut microbiota may modulate the production, metabolism, or signaling of serotonin (5-HT), dopamine, gamma-aminobutyric acid (GABA), histamine, and short-chain fatty acids, thereby potentially affecting intestinal barrier integrity, blood-brain barrier function, neuroinflammation, mood, and cognition (Qi et al., 2021; Koh et al., 2016; Chen M. et al., 2022; Bertollo et al., 2025). The hypothalamic-pituitary-adrenal (HPA) axis also provides a bidirectional endocrine route by which microbial signals may influence stress-related hormonal rhythms, whereas stress hormones can in turn affect intestinal permeability and microbial translocation (Bertollo et al., 2025; Jia et al., 2025; Tofani et al., 2025).

Immune signaling represents another major interface between the gut microbiota and the central nervous system (CNS). Peripheral cytokines and immune cells can communicate with CNS-associated compartments under physiological or pathological conditions, and gut-derived signals may shape microglial maturation, neuroinflammatory tone, and immune activity at CNS borders (Zierfuss et al., 2024; Sampson et al., 2016; Chen C. et al., 2022; Vara-Pérez and Movahedi, 2025). Experimental studies further suggest that gut-educated immune cells, including IgA-producing B cells and natural killer cells, may contribute to meningeal immune defense and inflammatory regulation, although the clinical relevance of these mechanisms requires further clarification (Fitzpatrick et al., 2020; Sanmarco et al., 2021).

**Figure 1:**
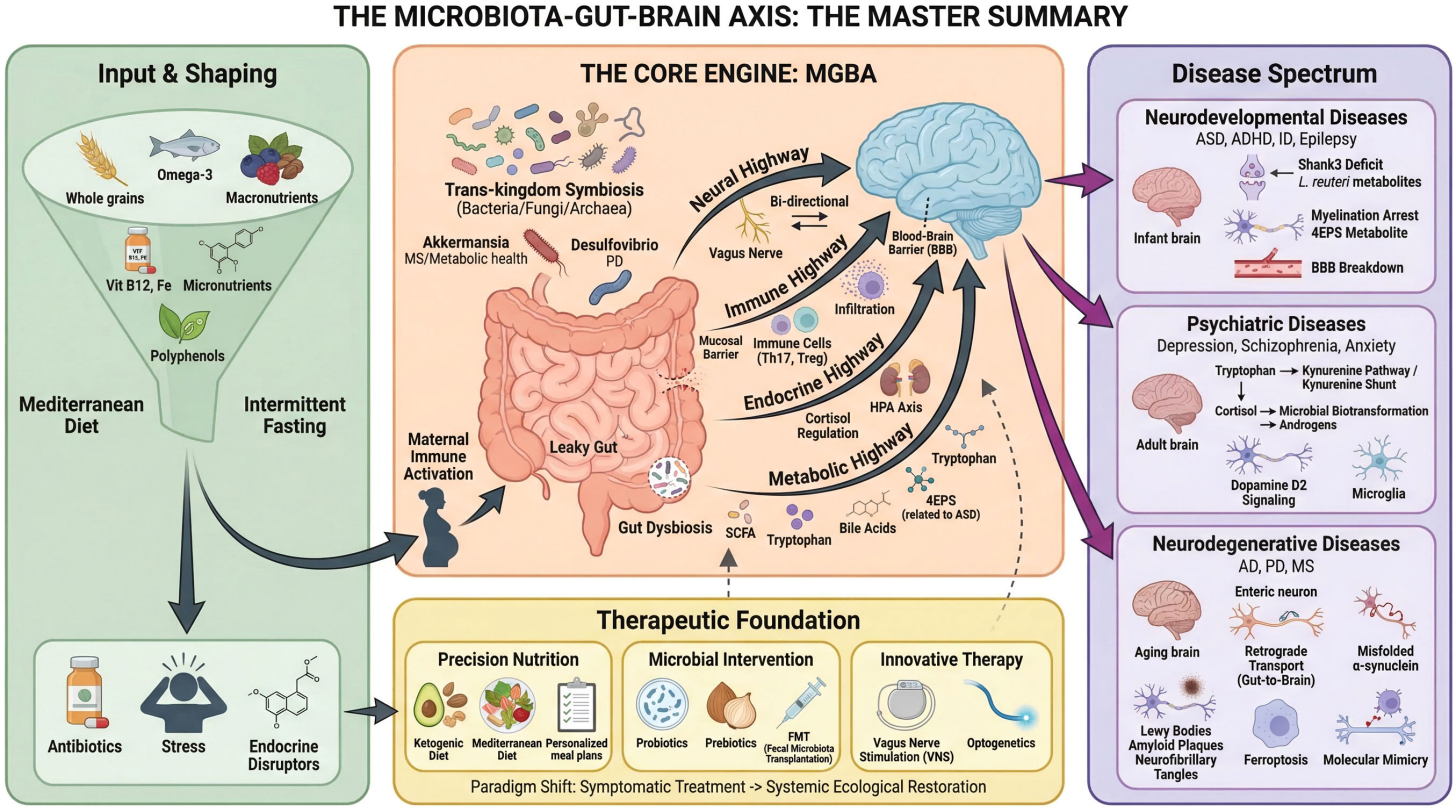
The microbiota-gut-brain axis as a systemic master engine mediating the interplay between environmental inputs and neuropsychiatric outcomes. Environmental factors, predominantly dietary patterns and early-life programming, dynamically shape the gut microenvironment. The MGBA orchestrates bidirectional communication via four primary highways: the neural network, the immune cascade, the endocrine system, and the metabolic pathway. Dysregulation within this trans-kingdom symbiosis-characterized by leaky gut and dysbiosis-propagates systemic signals that cross the blood-brain barrier, driving distinct pathological trajectories across the lifespan. These encompass neurodevelopmental, psychiatric, and neurodegenerative disorders. The paradigm shift toward systemic ecological restoration highlights targeted interventions, including precision nutrition, specific pre/probiotics, fecal microbiota transplantation, and innovative bioelectronic therapies.

Taken together, these findings indicate that the microbiota-gut-brain axis provides a useful framework for understanding how environmental factors, particularly diet, may influence neurodevelopment, neurological function, and vulnerability to neuropsychiatric disorders. This review therefore focuses on the interactions among diet, gut microbiota, and brain-related outcomes, with attention to mechanistic pathways, disease-associated evidence, therapeutic implications, and the challenges of translating preclinical findings into clinical practice. **Figure 1** and **Table 1** provides a schematic overview of these interconnected pathways.

**Table 1:** Summary of microbiota-related alterations, mechanisms, and representative interventions across neuropsychiatric disorders.

Disease / disorder	Key microbiota-related alterations	Proposed mechanisms	Representative interventions	Evidence level / notes
Alzheimer's disease	Dysbiosis associated with altered microbial metabolites; reduced intestinal *Bacteroides ovatus* and decreased lysophosphatidylcholine in 5xFAD models	Systemic inflammation, BBB dysfunction, microglial activation, Aβ deposition, Tau phosphorylation, mitochondrial dysfunction, oxidative stress, ferroptosis-related pathways	*B. ovatus* or LPC supplementation; *B. breve* MCC1274; diet-related microbiota modulation	Mainly preclinical mechanistic evidence; human causality and clinical efficacy remain insufficiently established
Parkinson's disease	Dysbiosis associated with gut-origin α-synuclein pathology; enrichment of *Desulfovibrio* reported in PD-related contexts; altered SCFA-producing taxa	Intestinal barrier dysfunction, enteric α-synuclein misfolding, vagal propagation, neuroinflammation, dopaminergic neuronal injury	Fecal microbiota transplantation; probiotics including *Bifidobacterium* and *Lactobacillus*; SCFA-supporting approaches	Evidence strongest for non-motor symptoms such as constipation; disease-modifying effects on motor symptoms remain unproven
Multiple sclerosis	Increased *Akkermansia muciniphila* in some cohorts; reduced Clostridia clusters XIVa and IV; altered butyrate-producing commensals	Peripheral immune activation, Th17/Treg imbalance, impaired immune tolerance, molecular mimicry, CNS autoimmunity, barrier dysfunction	Propionic acid; intermittent fasting; diet-based microbiota modulation	Supported by human cohort data, EAE models, and limited clinical intervention evidence; should be considered complementary to established immunomodulatory therapy
Depression	Dysbiosis associated with diet, stress, inflammatory comorbidities, and strain-specific immune effects; distinct *Bacteroides* strains may differentially regulate Th1/Th17 balance	Immune-inflammatory signaling, HPA-axis modulation, cortisol metabolism, dopamine D2 receptor-related signaling, tryptophan metabolism, NLRP3 inflammasome activation	Vagus nerve stimulation; Si-Ni-San; gingerol; milk fat globule membrane supplementation; probiotic or nutritional approaches	Evidence is heterogeneous; interventions differ in mechanism, target population, and clinical maturity
Schizophrenia	Cross-kingdom microbial alterations involving Archaea, Fungi, and Bacteria; antipsychotic-associated microbiota and metabolic changes	Neuroinflammation, dopaminergic signaling, host genome-microbiome interactions, mitochondrial and microbial metabolic signatures, gut-liver-brain interactions	Probiotics, prebiotics, antibiotics, FMT, dietary or vitamin-based approaches	Mostly preclinical or early translational evidence; microbiota-targeted strategies should be viewed as adjunctive and investigational
Anxiety disorders	Antibiotic-induced dysbiosis; altered SCFA-related metabolism; diet-associated dysbiosis such as malabsorbed fructose-related effects	Neuroimmune signaling, reduced intestinal acetylcholine, hippocampal microglial activation, microbial metabolite signaling, butyrate and inositol-related pathways	*Limosilactobacillus reuteri* NCU-37; heat-killed *Limosilactobacillus fermentum* PS150; aerobic exercise	Some randomized trial evidence exists, but strain specificity, placebo effects, population differences, and endpoint selection remain limitations
Intellectual disability	In Down syndrome, altered microbial profiles including increased *Parasporobacterium* and *Sutterella* and reduced SCFA-producing bacteria; reduced Acidaminococcaceae associated with cognitive scores	Immune dysregulation, reduced SCFA production, gut barrier changes, maternal microbiota-related neurodevelopmental programming, synaptic plasticity modulation	Probiotic-based modulation in experimental models; maternal diet-microbiota approaches	Human evidence remains mainly associative; dietary restriction, gastrointestinal symptoms, and medication exposure are important confounders
Autism spectrum disorder	Altered microbial developmental trajectories; reduced *Prevotella* in ASD with constipation; ASD-associated changes in microbial metabolic functions; altered 4EPS-related pathways	Neurotransmitter metabolism, tryptophan-serotonin pathways, myelination changes, maternal Th17/IL-17a signaling, immune-metabolic dysregulation	Microbiota transfer therapy; taurine and 5-aminovaleric acid supplementation; *Lactobacillus reuteri* in genetic models	Early clinical and preclinical evidence; larger randomized controlled trials with dietary control and standardized behavioral endpoints are needed
Attention-deficit/hyperactivity disorder	Microbiota alterations associated with inattention and hyperactivity; diet-related microbial and lipid metabolic changes; cross-species microbial correlates reported	Inflammatory signaling, neurotransmitter metabolism, tryptophan metabolism, microbiota-metabolism-behavior interactions	Kefir supplementation; psychobiotics; fermented foods; FMT under investigation	Early clinical evidence is emerging; FMT and psychobiotics remain investigational adjuncts
Epilepsy	Etiology-specific dysbiosis; *Lachnospira eligens* associated with BBB regulation; microbiota-sphingolipid and bile acid metabolic alterations	BBB dysfunction, neuroinflammation, altered neuronal excitability, sphingolipid metabolism, bile acid-HO-1-ferroptosis pathway, seizure threshold modulation	Ketogenic diet; metformin in experimental models; microbiome-informed response prediction; vagus nerve stimulation	Ketogenic diet is established for drug-resistant epilepsy, but microbiome changes may be causal mediators, biomarkers, or secondary effects

BBB, blood-brain barrier; FMT, fecal microbiota transplantation; MGBA, microbiota-gut-brain axis; SCFAs, short-chain fatty acids; Treg, regulatory T cell; Th17, T helper 17 cell; EAE, experimental autoimmune encephalomyelitis; LPC, lysophosphatidylcholine; 4EPS, 4-ethylphenylsulfate; HO-1, heme oxygenase-1.

## 2 Gut microbiota and diet

The gastrointestinal microbiota is established early in life and remains responsive to multiple host and environmental factors, among which diet is one of the most important modifiable influences (Beam et al., 2021). Dietary effects on the gut microbiota can be considered at several interconnected levels, including intestinal barrier integrity, microbial community structure, microbial metabolism, and immune regulation. Macronutrients provide substrates for microbial growth and fermentation, whereas specific dietary patterns can influence epithelial barrier function by regulating mucus production and tight junction-related proteins, such as occludin, claudins, and zonula occludens proteins. These changes may affect intestinal permeability, nutrient absorption, and mucosal immune defense (Zang et al., 2026). Such diet-barrier interactions may be particularly relevant during developmental windows, nutritional stress, or antibiotic exposure. For example, variation in dietary crude protein levels combined with antibiotic treatment has been reported to alter intestinal morphology and microbial diversity, with potential consequences for barrier defense and digestive function (Wang et al., 2025a). These findings suggest that diet contributes to host-microbe homeostasis not only by supplying microbial substrates but also by shaping the epithelial and immune interface of the gut.

Dietary patterns also influence systemic metabolic regulation by reshaping microbial communities and their functional outputs. Intermittent fasting, for example, has been associated with increased abundance of beneficial taxa, including *Akkermansia muciniphila*, and with induction of the microbial protein B2URF3, which has been implicated in vascular and metabolic regulation in experimental settings (Zeng et al., 2026). Evidence from cross-species and cross-population studies further suggests that diet-related microbial changes may participate in gut-heart and cardiometabolic interactions (Tang et al., 2026). Long-term dietary habits may also be associated with distinct enterotype patterns and metabolic susceptibility, indicating that sustained dietary exposure can influence both microbial ecology and host metabolic phenotype (Li et al., 2025b). Conversely, obesity-promoting dietary patterns may contribute to dysbiosis, low-grade inflammation, and altered estrogen metabolism, thereby linking diet-induced microbial imbalance with broader pathological processes. However, these associations are context-dependent, and their interpretation requires consideration of host genetics, baseline microbiota composition, medication use, and lifestyle factors.

In addition to macronutrients and overall dietary patterns, micronutrients and bioactive food components may influence the microbiota-gut-brain axis through metabolic and immunological mechanisms. Dietary tryptophan is a key example because its microbial and host-derived metabolites are involved in serotonin biosynthesis, kynurenine pathway activity, and central neurotransmission (Chehadi et al., 2026). Vitamin B12 may also affect microbial metabolic profiles and neurotransmitter-related pathways, suggesting that micronutrient status can modify the functional output of the gut microbiota (Xu et al., 2025a). Other dietary components, including vitamins, polyphenols, and essential metals, may contribute to immune homeostasis by altering microbial composition and inflammatory signaling (Rosso et al., 2026). Diets or supplements inspired by the Mediterranean dietary pattern have also been reported to reduce amyloid-related pathology and microglial activation in experimental models, supporting the possibility that diet-mediated microbial modulation may influence neuroinflammatory and neurodegenerative processes (Connell et al., 2026).

Despite these advances, the clinical translation of diet-microbiota-brain research remains challenging. Human dietary intake is difficult to standardize, and microbiota responses vary substantially across individuals because of differences in age, genetics, geography, medication exposure, disease status, and baseline microbial composition. Moreover, many mechanistic findings are derived from animal models or short-term intervention studies, whereas long-term human trials with standardized dietary assessment, microbiome profiling, metabolomics, and clinically meaningful neurological outcomes remain limited. Therefore, diet should be viewed as a promising but complex modulator of the microbiota-gut-brain axis rather than as a single deterministic factor. A more systematic integration of dietary patterns, microbial metabolites, barrier function, immune responses, and human clinical endpoints will be necessary to define how diet-base.

## 3 Gut microbiota and neurodegenerative and neuroinflammatory disorders

**Figure 2:**
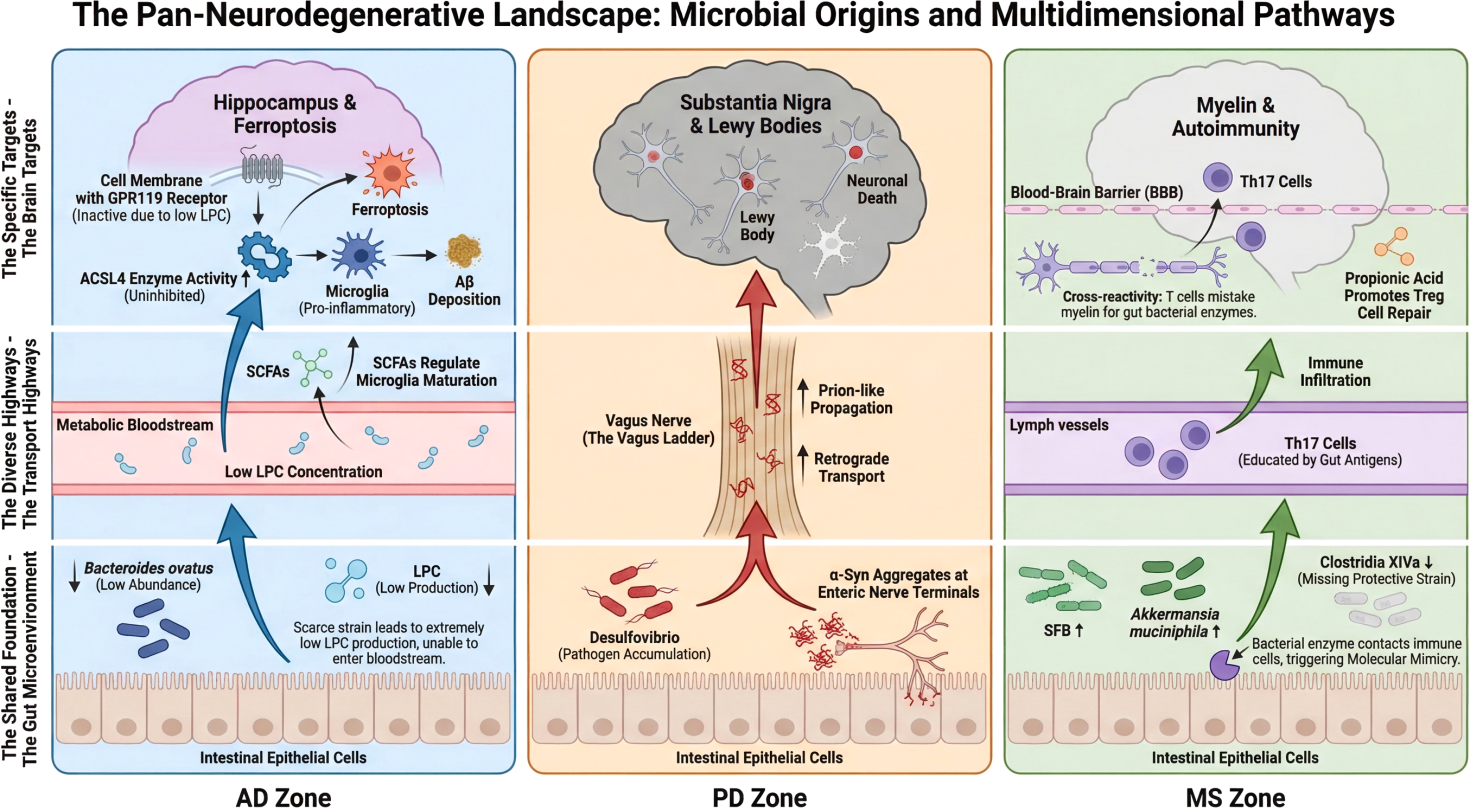
The pan-neurodegenerative landscape driven by distinct gut-origin microenvironmental disruptions. This comparative atlas delineates the multidimensional pathways linking specific microbial signatures to neurodegenerative pathologies. In the AD Zone, a reduced abundance of Bacteroides ovatus leads to a deficiency in circulating lysophosphatidylcholine. This scarcity fails to activate neuroprotective receptors, leaving the ACSL4 enzyme uninhibited, which subsequently triggers microglial pro-inflammatory responses, hippocampal Aβ deposition, and neuronal ferroptosis. In the PD Zone, the accumulation of specific pathogens initiates the misfolding and aggregation of α-synuclein at enteric nerve terminals. These aggregates undergo prion-like retrograde transport via the vagus nerve, ultimately forming Lewy bodies and causing dopaminergic neuronal death in the substantia nigra. In the MS Zone, an imbalance in commensal populations promotes molecular mimicry. Gut-educated Th17 cells cross-react with myelin autoantigens, migrating through lymphatic vessels to breach the BBB and drive central autoimmunity, while the loss of protective strains impairs propionic acid-mediated Treg cell repair.

Neurodegenerative disorders are characterized by progressive structural and functional impairment of specific neuronal populations within the central nervous system (CNS). Accumulating evidence suggests that gut dysbiosis may contribute to disease-related processes through the microbiota-gut-brain axis (MGBA), although the strength and direction of these associations vary across diseases and study models. Key mechanisms implicated in this interaction include neuroinflammatory signaling, microbial metabolite-mediated metabolic regulation, blood-brain barrier (BBB) dysfunction, and immune communication between the gut and CNS. This section focuses on representative neurodegenerative and neuroinflammatory conditions, including Alzheimer’s disease, Parkinson’s disease, and multiple sclerosis, while psychiatric and neurodevelopmental disorders are discussed separately in later sections (**Figure 2**).

### 3.1 Gut microbiota and Alzheimer’s disease

The emergence of high-throughput omics technologies has strengthened the observed association between gut dysbiosis and Alzheimer’s disease (AD). However, the gut microbiota should not be interpreted simply as either a secondary consequence or a direct cause of AD progression, because the direction and strength of this relationship may vary across experimental models and human cohorts. Current evidence suggests that dysbiotic microbial communities may contribute to AD-related pathological processes through several interconnected mechanisms, including systemic inflammation, blood-brain barrier (BBB) dysfunction, amyloid-beta (Aβ) deposition, and Tau phosphorylation (Zhang et al., 2023). Microglial activation is a major component of neuroinflammation and is closely associated with neuronal injury during AD progression (Qin et al., 2021). Experimental studies further indicate that the gut microbiota can regulate microglial maturation and immune homeostasis. For example, microglia from germ-free mice exhibit morphological and transcriptional abnormalities, whereas microbial metabolites such as short-chain fatty acids (SCFAs) can support microglial maturation and modulate CNS immune tone (Chen C. et al., 2022). Beyond neuroinflammation, gut-derived metabolites may also influence neuronal mitochondrial function, oxidative stress, and energy metabolism, thereby affecting pathways relevant to neurodegeneration (Ju et al., 2026). Other microbial metabolites, including secondary bile acids, trimethylamine N-oxide (TMAO), and tryptophan derivatives, may enter the circulation, cross or signal across the BBB, and interact with CNS pathways involved in neuronal function (Hou et al., 2023; Kang et al., 2024).

Microbiota-targeted approaches have therefore attracted increasing attention as potential adjunctive strategies for AD, although most mechanistic evidence remains preclinical. In germ-free conditions, mice carrying the high-risk ApoE4 background show reduced brain atrophy and neuroinflammation, suggesting that the gut microbiota may modulate ApoE4-associated glial metabolism and Tau-related neurodegeneration (Seo et al., 2023). In 5xFAD mice, reduced intestinal abundance of *Bacteroides ovatus* was associated with lower levels of its metabolite lysophosphatidylcholine (LPC). Supplementation with *B. ovatus* or LPC activated GPR119 signaling and suppressed ACSL4-related ferroptotic pathways, thereby reducing neuronal ferroptosis in this experimental model (Zha et al., 2025). Probiotic supplementation has also shown potential in preclinical AD models; for example, oral administration of *B. breve* MCC1274 increased brain-derived neurotrophic factor (BDNF), modulated Akt/GSK-3β signaling, reduced Tau hyperphosphorylation, improved Aβ-related cognitive impairment, and suppressed hippocampal amyloid production (Abdelhamid et al., 2022). Nevertheless, these findings should be interpreted cautiously. Evidence from animal models does not necessarily translate directly to human AD, and clinical application will require well-designed longitudinal studies that account for age, diet, medication use, disease stage, baseline microbiota composition, and cognitive outcomes.

### 3.2 Gut microbiota and Parkinson’s disease

Parkinson’s disease (PD) is a neurodegenerative disorder characterized clinically by bradykinesia, resting tremor, and rigidity, and pathologically by the abnormal accumulation of α-synuclein in the nervous system. Increasing evidence supports Braak’s “gut-origin hypothesis,” which proposes that, at least in a subset of patients, PD-related pathological changes may begin in the enteric nervous system (ENS) before spreading to the brain. Consistent with this concept, a multimodal imaging study identified a “body-first” subtype of PD in which early ENS impairment appears to precede overt brain pathology (Horsager et al., 2020).

Experimental studies provide mechanistic support for gut-to-brain propagation of α-synuclein pathology. Under conditions of intestinal barrier dysfunction and dysbiosis, misfolded α-synuclein may arise at enteric nerve terminals and spread retrogradely through the vagus nerve to the brainstem and substantia nigra, where it may contribute to dopaminergic neuronal injury. In mice, injection of pathological α-synuclein fibrils into the gut resulted in transmission to the brain through the vagus nerve, whereas truncal vagotomy prevented this pathological spread and attenuated PD-like neuropathology (Kim et al., 2019). In addition, enrichment of *Desulfovibrio* has been reported in PD-related contexts, and experimental evidence suggests that specific strains and their metabolites may promote α-synuclein aggregation, providing a possible microbial mechanism for dysbiosis-associated protein misfolding (Huynh et al., 2023).

Microbiota-targeted therapeutic approaches for PD, including fecal microbiota transplantation (FMT) and probiotic supplementation, aim to restore intestinal microbial balance and modulate MGBA-related immune and metabolic signaling. Available clinical and preclinical evidence suggests that these interventions may increase short-chain fatty acid-producing taxa, including *Prevotella* and *Blautia*, improve intestinal barrier function, and reduce systemic pro-inflammatory cytokines such as TNF-α and IL-6 (Tan et al., 2021; Kuai et al., 2021). Clinically, these approaches appear most promising for non-motor symptoms, particularly constipation, and may also have potential effects on sleep disturbance and anxiety. However, current evidence remains insufficient to establish durable disease-modifying effects on core motor symptoms or dopaminergic neurodegeneration. Larger controlled trials with longer follow-up, standardized microbiome endpoints, and clinically meaningful motor and non-motor outcomes are needed before microbiota-targeted interventions can be considered established therapies for PD.

### 3.3 Gut microbiota and multiple sclerosis

Multiple sclerosis (MS) is an immune-mediated demyelinating and neuroinflammatory disease of the central nervous system. The relationship between the gut microbiota and MS has become an important topic in neuroimmunology because clinical and experimental studies suggest that gut dysbiosis may influence peripheral immune activation and CNS autoimmunity. However, the strength of this relationship varies across cohorts and models, and causality should be interpreted with caution. Clinical cohort studies have reported altered gut microbial profiles in individuals with MS, including increased abundance of *Akkermansia muciniphila* in some cohorts (iMSMS Consortium, 2022). Although this bacterium is often considered beneficial in metabolic contexts, its enrichment in MS has been associated with host immune activation, suggesting that microbial effects may be disease- and context-dependent (Jangi et al., 2016). Conversely, reductions in protective commensals, particularly members of Clostridia clusters XIVa and IV, may decrease intestinal butyrate production, weaken barrier integrity, and impair immune tolerance (Miyake et al., 2015).

Experimental studies have provided mechanistic insight into how the gut microbiota may shape CNS autoimmunity. Germ-free mice show reduced susceptibility to experimental autoimmune encephalomyelitis (EAE), supporting a role for commensal microbiota in promoting autoimmune responses under specific experimental conditions (Berer et al., 2021). Cross-species transplantation studies further suggest that microbiota from MS patients can transfer disease-associated immune phenotypes to germ-free mice, promoting pro-inflammatory T-cell differentiation and increasing EAE severity (Correction for Cekanaviciute et al. Gut bacteria from multiple sclerosis patients modulate human T cells and exacerbate symptoms in mouse models, 2017). These findings support a potential contributory role for dysbiosis in MS-related immune dysregulation, while also emphasizing the need to distinguish experimental causality from direct clinical causality in human disease.

At the immune-mechanistic level, dysbiosis may influence the balance between pro-inflammatory and regulatory immune cell populations. Certain microbial antigens, including those derived from segmented filamentous bacteria, can promote pathogenic Th17-cell differentiation, and these cells may subsequently enter the CNS and contribute to demyelinating inflammation (Ivanov et al., 2009). In contrast, reduced abundance of Clostridia-related commensals may impair regulatory T-cell (Treg) induction, weakening peripheral immune tolerance and favoring excessive immune activation (Atarashi et al., 2013). Molecular mimicry has also been proposed as a possible mechanism linking gut microbes to CNS autoimmunity. For example, some microbial enzymes, including GDP-L-fucose synthase, show structural similarity to CNS myelin antigens and may activate cross-reactive autoreactive T cells in susceptible contexts (Planas et al., 2018).

Given these mechanisms, microbiota-targeted and metabolite-based interventions have attracted interest as adjunctive strategies for MS. Supplementation with short-chain fatty acids, particularly propionic acid, has been reported to improve Treg-cell function in MS patients and has been associated with reductions in annual relapse rate and brain atrophy progression (Duscha et al., 2020). Lifestyle and dietary interventions may also influence the gut microbiota. For example, intermittent fasting has been shown in experimental models to alter microbial composition and increase metabolites with immunomodulatory and neuroprotective properties, thereby attenuating CNS autoimmune responses and promoting remyelination (Cignarella et al., 2018). Nevertheless, these approaches should be interpreted as complementary rather than replacement strategies for established immunomodulatory therapies. Their clinical application requires further validation through controlled human studies that define optimal dosing, treatment duration, patient selection, microbiome biomarkers, and long-term safety.

## 4 Gut microbiota and psychiatric disorders

**Figure 3:**
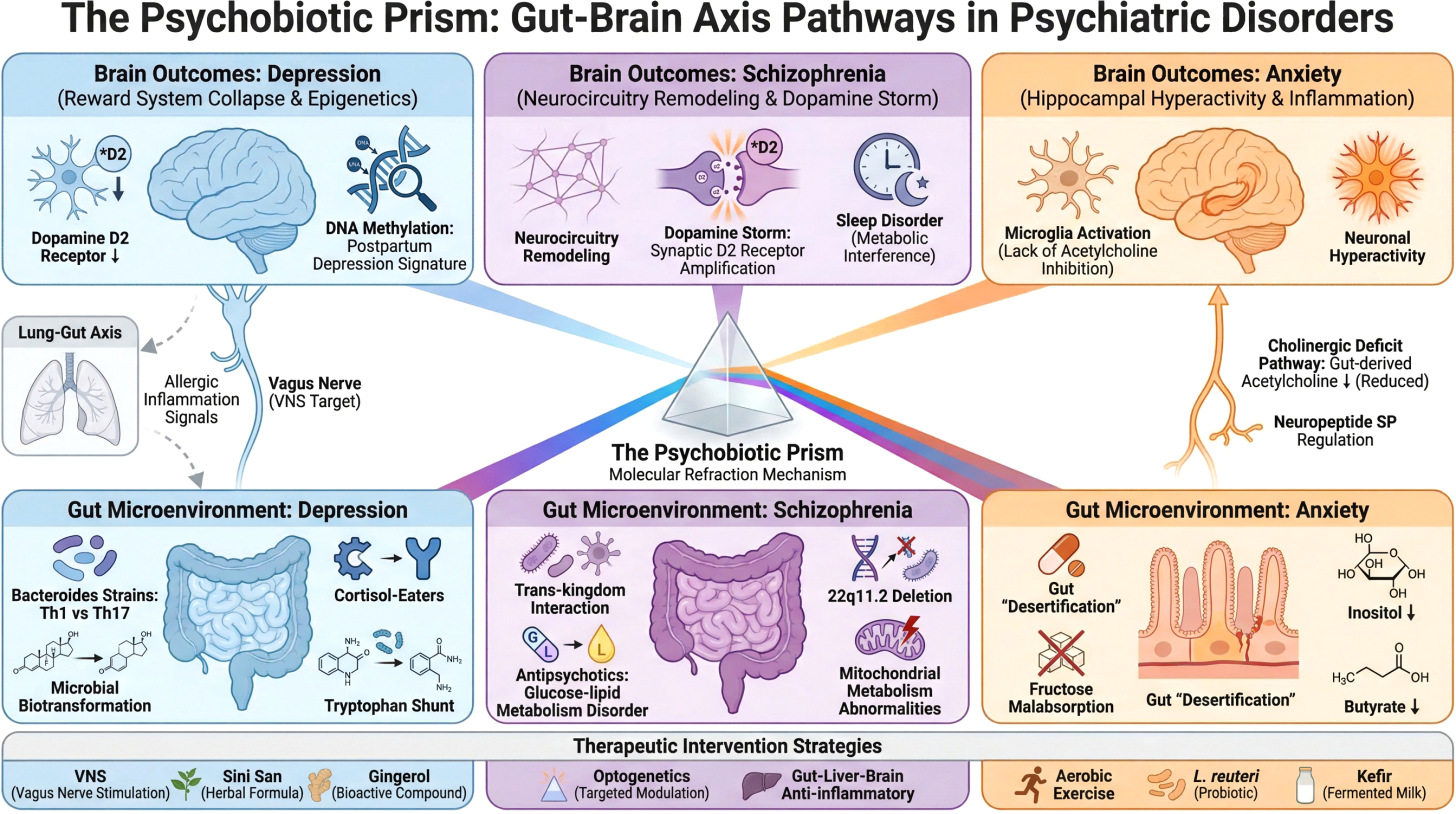
The psychobiotic prism: refraction of gut microenvironmental shifts into specific psychiatric phenotypes. The gut ecosystem acts as a metabolic and immune “prism,” differentially refracting systemic signals to drive distinct psychiatric outcomes. In Depression, cortisol-metabolizing bacteria and tryptophan shunting disrupt central neurotransmitter biosynthesis, leading to reward system collapse and specific epigenetic modifications. In Schizophrenia, trans-kingdom microbial interactions and mitochondrial metabolic abnormalities amplify synaptic D2 receptor signaling and drive neurocircuitry remodeling, processes further complicated by antipsychotic-induced glucose-lipid metabolic disorders. In Anxiety, gut “desertification” and reduced microbial metabolites precipitate a gut-derived cholinergic deficit. This reduction in acetylcholine signaling disinhibits microglial activation, culminating in hippocampal hyperactivity and neuroinflammation. Targeted therapeutic strategies—ranging from herbal formulas and bioactive compounds to specific probiotics and aerobic exercise—aim to restore this neuro-microbial homeostasis.

Psychiatric disorders have traditionally been conceptualized primarily as disorders of the central nervous system (CNS). However, accumulating evidence from nutritional psychiatry and microbiome research suggests that the gut ecosystem may influence mood, cognition, and behavior through the microbiota-gut-brain axis (MGBA). This bidirectional communication involves neural pathways, including the vagus nerve, endocrine signaling through the hypothalamic-pituitary-adrenal axis, immune mediators such as cytokines, and microbial metabolic products. Current clinical and experimental evidence supports an association between dysbiosis and psychiatric disorders such as depression, schizophrenia, and anxiety disorders. Nevertheless, dysbiosis should not be interpreted as a uniform or independent cause of psychiatric illness, because microbial changes may reflect disease-related behavior, diet, medication exposure, environmental stress, or comorbid somatic conditions. The following subsections summarize disease-specific evidence and highlight the need to distinguish mechanistic hypotheses from clinically established causality (**Figure 3**).

### 4.1 Depression

The relationship between gut microbiota and depression involves immune, endocrine, metabolic, and environmental components. Recent studies suggest that broad taxonomic generalizations may be insufficient, as different strains within the same genus can exert distinct immunological effects. For example, different *Bacteroides* strains may differentially regulate intestinal Th1/Th17-cell balance, thereby influencing CNS immune tone and depressive-like behaviors in experimental settings (Li et al., 2026c). Diet-microbiota-immune interactions may also contribute to emotional homeostasis. Conversely, maladaptive dietary patterns may promote dysbiosis and immune dysregulation, potentially increasing susceptibility to depression-related phenotypes (Marano et al., 2025). Inflammatory comorbidities provide another relevant context. In models of chronic allergic pulmonary inflammation, lung-gut interactions have been associated with depressive-like behaviors, suggesting that systemic inflammatory diseases may influence mood through microecological mechanisms (Kanaya et al., 2025). Depression-associated dysbiosis has also been linked to immune-mediated liver injury through NLRP3 inflammasome activation, indicating that gut-brain interactions may occur within broader multi-organ inflammatory networks (Zhou et al., 2026). In addition, host-microbiome immune biomarkers are being investigated as potential indicators of stress-related depression, although their clinical validity requires further confirmation (Zhang et al., 2026).

Gut microbiota may also participate in neuroendocrine and neurotransmitter-related pathways relevant to depression. Certain gut bacteria possess enzymatic systems capable of converting cortisol into androgenic metabolites, suggesting a possible microbial route for modifying stress hormone metabolism (Wang X. et al., 2026). The microbiota may also influence dopaminergic signaling, including dopamine D2 receptor-related pathways, thereby affecting reward-related circuitry (Xie et al., 2025). Vitamin B12 metabolism represents another potential interface between microbiota and neurotransmitter biosynthesis (Xu et al., 2025b). In parallel, tryptophan metabolism links dietary intake, microbial metabolism, serotonin biosynthesis, and kynurenine pathway activity, providing a mechanistic bridge between nutrition, microbiota, and mood regulation (Chehadi et al., 2026). Studies of postpartum depression further suggest that host-microbe metabolic interactions may be associated with changes in circulating metabolites and DNA methylation patterns, supporting the potential use of microbial metabolites as biomarkers, while also requiring validation in larger clinical cohorts (Zhang et al., 2026).

Depression often occurs in the context of environmental exposure, chronic stress, or somatic comorbidity, and the gut microbiota may act as one mediator within this broader network. Environmental toxicology studies suggest that endocrine-disrupting chemicals may perturb gut microbial homeostasis and neuroendocrine signaling, thereby contributing to mood-related abnormalities (Uğur, 2025). Chronic environmental stress in mice has been associated with time-dependent changes in gut microbiota and multi-system dysfunction, including emotional phenotypes (Qiao et al., 2026). In clinical comorbidities, specific gut microbial signatures have been associated with psychological distress in fibromyalgia, suggesting a possible role of microbiota in pain-mood interactions (Lanzara et al., 2026). Among cancer survivors, particularly melanoma survivors receiving immunotherapy, long-term psychological symptoms have been linked to dietary habits and gut microbiome composition, suggesting that microbiota-related factors may represent modifiable targets for supportive care (Robert et al., 2025).

Several gut-brain-directed interventions have been explored for depression, including neuromodulation, pharmacological approaches, dietary strategies, and probiotic or nutritional supplementation. Vagus nerve stimulation (VNS), an established intervention for treatment-resistant depression, may partly act through microbiota remodeling and tryptophan metabolism regulation. Multi-omics analyses support a possible neuro-microbial mechanism for VNS-related effects, and clinical studies suggest potential relevance for gut-brain axis disorders (Ning et al., 2025; Pan et al., 2026). Traditional Chinese medicine formulations such as Si-Ni-San have been reported in randomized clinical trials to reduce breast cancer-related depression, potentially through systemic regulation of gut-brain signaling (Hong et al., 2026). Bioactive plant compounds such as gingerol may also exert anxiolytic or antidepressant-like effects by modulating colonic microbiome-associated neuroimmune responses (Mendóza et al., 2026). Nutritional interventions, including milk fat globule membrane supplementation, have shown beneficial effects in stress-related experimental depression models by restoring microbial balance and reducing neuroinflammation (Huang et al., 2026a). However, these interventions differ substantially in evidence level, mechanism, target population, and clinical maturity. Larger controlled trials with standardized psychiatric endpoints, dietary assessment, microbiome profiling, and long-term follow-up are needed before microbiota-targeted strategies can be considered established treatments for depression.

### 4.2 Schizophrenia

Schizophrenia is a severe psychiatric disorder involving complex interactions among genetic susceptibility, neurodevelopmental processes, immune signaling, neurotransmitter systems, and environmental factors. Recent microbiome studies suggest that schizophrenia may be associated with cross-kingdom microbial alterations involving Archaea, Fungi, and Bacteria, rather than changes in bacterial communities alone. These microbial changes may contribute to immune and metabolic dysregulation, but they should not be interpreted as definitive causal drivers of schizophrenia without further validation (Fu et al., 2026). Neuroinflammation has been proposed as one mechanism linking gut dysbiosis with CNS dysfunction, as gut-derived inflammatory signals may influence the brain immune environment and potentially affect neural circuit function (Nayak et al., 2026).

At the neurotransmitter level, the gut microbiota may influence dopaminergic pathways relevant to schizophrenia, including dopamine D2 receptor-related signaling (Xie et al., 2025). This provides a peripheral microecological perspective on the dopamine hypothesis of schizophrenia, although the clinical significance of this mechanism remains to be clarified. Microbiota-related changes may also interact with comorbid symptoms. For example, in schizophrenia patients with sleep disturbance, alterations in gut taxa and metabolic profiles have been associated with neurotransmission-related pathways, suggesting a possible gut-microbiota-sleep-brain interaction (Huang et al., 2026b).

Host genetic susceptibility may further shape microbiota-related metabolic features. In 22q11.2 deletion syndrome, a genetic condition associated with increased psychosis risk, urinary metabolomic analyses have identified microbial and mitochondrial metabolic signatures correlated with psychosis-related traits (Minami et al., 2025). Molecular studies also suggest that long noncoding RNAs may participate in schizophrenia-related pathways by interacting with gut microbial and host immune-metabolic networks (Dang et al., 2026). These findings support the concept of host genome-microbiome interactions, but additional mechanistic and longitudinal studies are needed to determine whether these signatures are causal contributors, compensatory responses, or disease-associated biomarkers.

Clinical treatment and environmental exposure add further complexity to microbiota-schizophrenia associations. Second-generation antipsychotics can alter gut microbiota composition and serum metabolic profiles even during early treatment, suggesting that drug-induced microecological remodeling may contribute to glucose and lipid metabolic side effects (Wu et al., 2025). Environmental factors, including endocrine-disrupting chemicals, may also perturb gut microecology and neuroendocrine signaling, potentially modifying psychiatric vulnerability through microbiota-related pathways (Uğur, 2025). Nutritional interventions, including vitamin supplementation, have therefore attracted interest because vitamins and gut microbiota can interact bidirectionally during brain development and metabolic regulation (Uğur, 2025).

Microbiota-targeted interventions for schizophrenia, including probiotics, prebiotics, antibiotics, and fecal microbiota transplantation (FMT), are currently best viewed as investigational or adjunctive approaches rather than replacements for established psychopharmacological treatment. Preclinical and early translational studies suggest that these interventions may modulate host neurochemistry and immune signaling and improve behavioral or biochemical abnormalities in experimental models (Fond et al., 2020; Borkent et al., 2022). However, clinical evidence remains limited, and the heterogeneity of schizophrenia, antipsychotic exposure, diet, metabolic status, and baseline microbiota composition complicates interpretation (Zhao et al., 2024; Dziedziak et al., 2025). The gut-liver-brain axis has also been proposed as a framework linking microbial inflammation, hepatic metabolic function, and CNS immune activation (Aghara et al., 2025). In addition, emerging approaches such as optogenetic modulation have been discussed in experimental contexts, but their clinical relevance to microbiota-based schizophrenia treatment remains speculative at present (Patrono et al., 2021). Future studies should clarify patient selection, intervention timing, safety, and clinically meaningful psychiatric outcomes before these strategies can be incorporated into routine care.

### 4.3 Anxiety disorders

Anxiety disorders are increasingly being studied within a multidimensional framework that includes gut microbiota, immune regulation, neuroendocrine signaling, and microbial metabolism. Preclinical models suggest bidirectional interactions between gastrointestinal dysfunction and anxiety-like behavior, but direct causality in human anxiety disorders remains insufficiently established (Wang Y-H. et al., 2025). Neuroimmune mechanisms may represent one important link. For example, antibiotic-induced dysbiosis has been associated with sustained reductions in intestinal acetylcholine, hippocampal microglial activation, and anxiety-like behavior in experimental models, suggesting that cholinergic signaling may be one microbiota-related pathway influencing emotional regulation (Xu et al., 2026). In inflammatory contexts such as colitis and neuropathic pain, anxiety-like behaviors may also be modulated by gut microbial changes and downstream metabolites. Neuropeptide-related pathways and phytochemicals such as gingerol have been implicated in microbiota-associated neuroimmune regulation (Lan et al., 2026; Mendóza et al., 2026).

Dietary and metabolic factors also contribute to microbiota-anxiety interactions. Dietary patterns may influence psychological states by modulating immune-microbiota-brain communication (Marano et al., 2025). Certain dietary components, such as malabsorbed fructose, have been reported to induce dysbiosis and anxiety-like behavior in experimental settings, supporting a potential role for metabolic disturbance in emotional regulation (Coursan et al., 2026). Conversely, microbial metabolites such as butyrate and inositol may exert neuroprotective or anxiolytic-like effects by modulating neuroinflammation and neurotransmitter balance (Korenblik et al., 2026; Lan et al., 2026). These findings support a diet-microbiota-metabolism framework for anxiety, although human evidence remains heterogeneous and requires more controlled validation.

Clinical intervention strategies for anxiety are expanding beyond conventional pharmacotherapy to include probiotics, paraprobiotics, and lifestyle interventions. Randomized controlled trials have reported that *Limosilactobacillus reuteri* NCU-37 may reduce anxiety symptoms in perimenopausal women through microbiota remodeling (Wu et al., 2026). Heat-killed *Limosilactobacillus fermentum* PS150 has also been associated with improved sleep quality and reduced anxiety severity, suggesting that paraprobiotics may influence mental states (Lee et al., 2025). Aerobic exercise represents another non-pharmacological intervention that may reduce anxiety by increasing short-chain fatty acid-producing bacteria and modulating gut-brain metabolic signaling (Zheng et al., 2025). Nevertheless, strain specificity, population differences, trial duration, placebo effects, and endpoint selection remain important limitations. Further human studies are needed to determine which patients are most likely to benefit from microbiota-targeted or lifestyle-based interventions.

## 5 Gut microbiota and neurodevelopmental disorders

**Figure 4:**
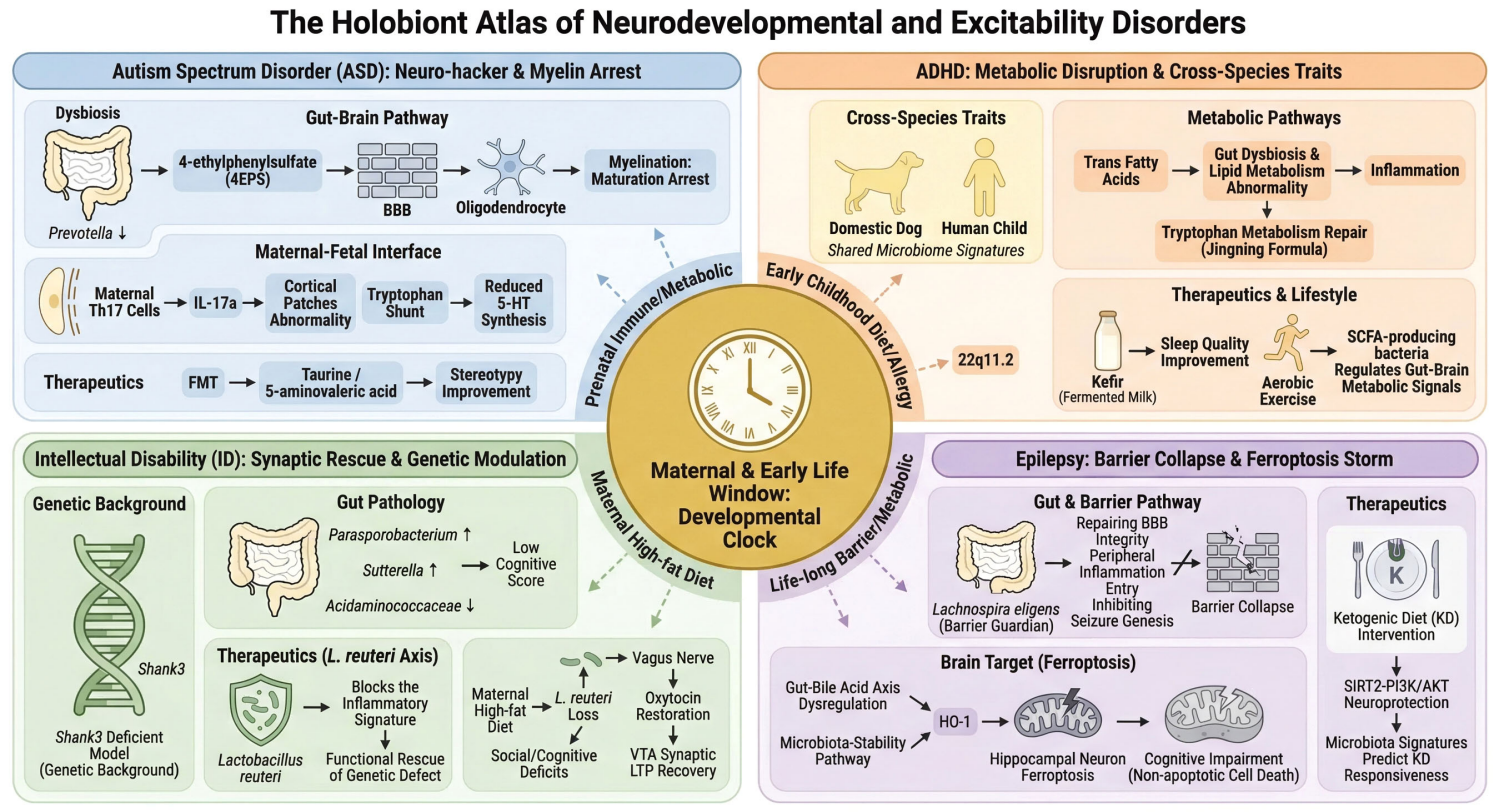
The holobiont atlas of neurodevelopmental and excitability disorders, emphasizing the maternal-fetal interface and critical developmental windows. Early-life microbial programming profoundly dictates central nervous system trajectory. In Autism Spectrum Disorder, maternal Th17-derived IL-17a traverses the placenta, altering cortical patch formation, while postnatal dysbiosis elevates neurotoxic metabolites like 4-ethylphenylsulfate, which breaches the BBB to arrest oligodendrocyte myelination. In Intellectual Disability, even against a definitive genetic background, an altered gut inflammatory signature exacerbates cognitive decline; notably, intervention with Lactobacillus reuteri can functionally rescue synaptic long-term potentiation in the ventral tegmental area via the vagus-oxytocin pathway. In Attention-Deficit/Hyperactivity Disorder, dietary triggers disrupt lipid metabolism and tryptophan pathways, driving systemic inflammation that mirrors cross-species behavioral traits. In Epilepsy, the loss of barrier-guarding commensals precipitates BBB collapse and peripheral immune infiltration. Concurrently, dysregulation of the gut-bile acid axis induces HO-1-dependent hippocampal ferroptosis. Interventions such as the Ketogenic Diet offer neuroprotection by modulating specific microbiota signatures and activating the SIRT2-PI3K/AKT pathway.

Neurodevelopmental disorders have traditionally been interpreted primarily through genetic and central nervous system-based mechanisms. Increasing evidence, however, suggests that the microbiota-gut-brain axis (MGBA) may act as an environmental modifier of neurodevelopment by influencing immune maturation, microbial metabolite production, vagal signaling, and synaptic plasticity. This framework does not replace established genetic and neurobiological models but provides an additional perspective for understanding how early-life microbial, dietary, and immune exposures may shape developmental trajectories. In this section, we discuss representative neurodevelopmental disorders, including intellectual disability, autism spectrum disorder, and attention-deficit/hyperactivity disorder. Epilepsy is discussed separately because it is more appropriately considered an excitability-related neurological disorder rather than a core neurodevelopmental category (**Figure 4**).

### 5.1 Gut microbiota and intellectual disability

Intellectual disability (ID) is a heterogeneous condition with diverse genetic, developmental, metabolic, and environmental contributors. Although genetic factors play a central role in many forms of ID, emerging evidence suggests that the gut microbiota may modify neurodevelopmental and cognitive outcomes through immune, metabolic, and barrier-related mechanisms. In Down syndrome (DS), one of the most common genetic conditions associated with ID, early studies identified an altered gut microbial profile characterized by increased abundance of genera such as *Parasporobacterium* and *Sutterella*, together with reduced short-chain fatty acid-producing bacteria (Biagi et al., 2014). Recent clinical studies further linked microbial alterations to cognitive phenotypes. For example, Ren et al. reported reduced abundance of Acidaminococcaceae in Chinese children with DS and found associations between microbial structure and lower cognitive function scores, suggesting that dysbiosis may contribute to, or reflect, cognitive impairment in this population (Ren et al., 2022). However, causal interpretation requires caution. Large-scale metagenomic evidence indicates that restrictive dietary preferences, including picky eating, may partly explain reduced microbial diversity in neurodevelopmental disorders. These findings highlight the need to adjust for dietary habits, medication exposure, gastrointestinal symptoms, and other confounders when interpreting microbiota-cognition associations (Yap et al., 2021).

Maternal and early-life microbial environments may also influence neurodevelopment during critical windows. Experimental evidence suggests that metabolites produced by the maternal gut microbiota can cross the placental interface and influence fetal thalamocortical axon development and sensory processing (Vuong et al., 2020). Maternal diet may further modify offspring neurodevelopment through microbiota-dependent mechanisms. For example, maternal high-fat diet has been associated with reduced offspring intestinal abundance of *Lactobacillus reuteri*, lower oxytocin signaling in the ventral tegmental area, impaired synaptic plasticity, and social or cognitive abnormalities in experimental models (Buffington et al., 2016). These findings support a possible maternal diet-microbiota-neurodevelopment pathway, although their relevance to human ID requires further validation.

Gut barrier integrity and microbial metabolites provide additional mechanisms through which gut dysbiosis may influence neurodevelopmental phenotypes. In maternal immune activation models, intestinal barrier defects increase systemic exposure to microbial metabolites such as 4-ethylphenylsulfate, which has been linked to anxiety-like behavior. Oral administration of the human commensal *Bacteroides fragilis* improved barrier function and behavioral abnormalities in this experimental context (Hsiao et al., 2013). In genetic models, microbiota-based interventions may also modulate synaptic plasticity. In the *Shank3* deletion model, probiotic supplementation restored long-term potentiation in the ventral tegmental area through the vagus nerve-oxytocin pathway, suggesting that gut-derived signals can partially modify circuit dysfunction even in the presence of genetic vulnerability (Sgritta et al., 2019). Nevertheless, these findings remain largely preclinical and should be interpreted as evidence for potential modulation rather than definitive clinical rescue.

### 5.2 Gut microbiota and autism spectrum disorder

Autism spectrum disorder (ASD) is a heterogeneous neurodevelopmental condition influenced by genetic, environmental, immune, metabolic, and behavioral factors. Rather than viewing ASD solely as a CNS-restricted disorder, recent microbiome research has suggested that gut microbial composition and function may be associated with gastrointestinal symptoms, immune activation, metabolic alterations, and behavioral phenotypes in some individuals with ASD. However, the direction of causality remains complex because restricted diet, gastrointestinal dysfunction, medication exposure, and behavioral patterns may also shape the gut microbiota.

Multi-omics studies have provided broader insight into ASD-associated microbiota patterns. Morton et al. integrated microbiomic, metabolomic, and brain transcriptomic datasets and identified microbial taxa, including *Prevotella* and *Bifidobacterium*, as well as microbial metabolic functions related to amino acid and carbohydrate metabolism that were associated with brain gene expression and peripheral inflammatory profiles (Morton et al., 2023). Additional studies suggest that children with ASD, especially those with constipation, may show altered microbial developmental trajectories and reduced abundance of genera such as *Prevotella*. These microbial shifts have been associated with changes in neurotransmitter-related metabolic pathways, including GABA, serotonin, and dopamine metabolism (Dan et al., 2020). Importantly, large-scale metagenomic studies indicate that restrictive dietary preferences may be a major contributor to reduced microbial diversity in ASD, underscoring the need to distinguish disease-associated dysbiosis from diet-driven microbial changes (Yap et al., 2021).

Experimental studies have identified several candidate microbial mechanisms relevant to ASD-like phenotypes. Gut-derived 4-ethylphenylsulfate has been reported to accumulate in ASD-related models and may influence oligodendrocyte maturation, myelination, functional connectivity, and anxiety-like behavior (Needham et al., 2022). Cross-species microbiota transplantation studies further suggest that microbiota from children with ASD can alter colonic serotonin synthesis and brain tryptophan metabolism in germ-free mice, producing autism-like behavioral phenotypes in experimental settings (Xiao et al., 2021). Maternal immune-microbiota interactions may also contribute to early neurodevelopmental vulnerability. Specific maternal gut bacteria can promote Th17-cell differentiation and IL-17a production, which may affect fetal cortical development in maternal immune activation models (Kim et al., 2017).

Microbiota-targeted interventions have shown potential in experimental and early clinical studies, but their clinical interpretation requires caution. Human-to-mouse fecal microbiota transplantation studies suggest that ASD-associated microbiota can transfer behavioral features and that supplementation with metabolites such as taurine and 5-aminovaleric acid may improve repetitive behaviors in mice (Sharon et al., 2019). In the *Shank3* deletion model, supplementation with *Lactobacillus reuteri* restored ventral tegmental area synaptic plasticity through vagus nerve-oxytocin signaling, supporting a possible microbiota-circuit interaction in genetically vulnerable contexts (Sgritta et al., 2019). Clinical follow-up studies of microbiota transfer therapy reported sustained improvements in gastrointestinal and behavioral symptoms in children with ASD up to two years after treatment (Kang et al., 2019). However, these findings require replication in larger randomized controlled trials with standardized behavioral endpoints, dietary control, microbiome profiling, safety monitoring, and careful patient stratification.

### 5.3 Gut microbiota and attention-deficit/hyperactivity disorder

Attention-deficit/hyperactivity disorder (ADHD) is increasingly being investigated within a microbiota-gut-brain framework. Current evidence suggests that gut microbial alterations may be associated with inflammatory signaling, neurotransmitter metabolism, and behavioral traits related to inattention and hyperactivity, although direct causality in human ADHD remains insufficiently established (Yuan et al., 2025). Cross-species observations provide additional support for a microbiota-behavior association. For example, dogs with inattentive and hyperactive-impulsive behaviors have been reported to show gut microbiome features resembling those observed in human ADHD, suggesting that microbial correlates of hyperactivity and inattention may be partially conserved across species (Salamon et al., 2025). These findings support further investigation of microbiota-related mechanisms in pediatric neurodevelopmental disorders but should be interpreted cautiously in relation to clinical causality (AlKuwaiti et al., 2025).

Dietary patterns may be particularly relevant to ADHD because diet can shape microbial composition, lipid metabolism, inflammatory tone, and neurotransmitter-related pathways. Pro-inflammatory dietary components, including trans fatty acids, have been associated with altered gut microbial diversity and lipid metabolic pathways, which may contribute to inflammatory signaling and ADHD-related symptom severity (He et al., 2025). Conversely, interventions that restore microbial and metabolic balance may have therapeutic potential. For example, the traditional Chinese medicine Jingning improved ADHD-like behaviors in animal models, possibly by restoring gut microbial balance and tryptophan metabolism (Yang et al., 2026). These findings suggest that the microbiota-metabolism-behavior axis may be relevant to ADHD, but the evidence remains heterogeneous and requires further clinical validation.

Microbiota-targeted strategies for ADHD are moving from exploratory research toward early clinical evaluation. Psychobiotics, dietary supplementation, fermented foods, and fecal microbiota transplantation (FMT) are being investigated as possible adjunctive approaches. In one randomized controlled trial, kefir supplementation in children with ADHD altered gut microbial composition and was associated with improved sleep quality and reduced hyperactivity and inattention symptoms (Lawrence et al., 2025). FMT has also been proposed as a potential intervention for complex or refractory cases, aiming to reconstruct gut microbial ecology and gut-brain signaling (Xiao et al., 2025). However, FMT for ADHD remains experimental, and its long-term safety, donor selection, optimal timing, durability, and psychiatric efficacy require rigorous evaluation. At present, microbiota-targeted strategies should be considered investigational adjuncts rather than established replacements for conventional ADHD management.

## 6 Gut microbiota and epilepsy

Epilepsy is a neurological disorder characterized by recurrent seizures and abnormal neuronal excitability. Although epilepsy is not uniformly classified as a neurodevelopmental disorder, gut microbiota-related mechanisms may influence seizure susceptibility, neuroinflammation, blood-brain barrier (BBB) function, and cognitive comorbidities. Therefore, epilepsy is more appropriately discussed as an excitability-related neurological condition within the broader MGBA framework.

Gut microbiota may contribute to epilepsy-related processes through barrier regulation and immune homeostasis. Certain commensal bacteria have been proposed to act as peripheral regulators of BBB integrity. For example, *Lachnospira eligens* has been reported to improve BBB dysfunction and reduce neuroinflammatory responses in early-stage epilepsy models (Li et al., 2026a). Dysbiosis may also vary according to epilepsy etiology. In tuberous sclerosis complex-related epilepsy, comparative analyses suggest both shared and etiology-specific microbial signatures, indicating that microbiota changes may reflect disease background, seizure activity, medication exposure, or host metabolic state rather than a uniform epilepsy-associated pattern (Ottaviano et al., 2025).

Microbial metabolites may also influence neuronal excitability and survival-related pathways. Multi-omics analyses have implicated a microbiota-sphingolipid metabolic axis in generalized epilepsy, suggesting that microbial regulation of sphingolipid metabolism may affect neuronal membrane stability and seizure susceptibility (41395464). Disruption of the gut microbiota-bile acid axis has also been linked to heme oxygenase-1-dependent ferroptosis and hippocampal neuronal injury, providing a potential mechanism for epilepsy-associated cognitive impairment (Li et al., 2025a).

The gut microbiota-neuroinflammation axis may further connect epilepsy with common neuropsychiatric comorbidities, including depression and cognitive deficits. Dysbiosis-associated inflammatory signaling may influence CNS inflammatory tone and seizure threshold, although the direction and magnitude of these effects remain model-dependent (Wang X. et al., 2025). Metabolic regulators are also being reconsidered in this context. For example, metformin has been reported to reshape gut microbiota and inhibit the TLR4/MyD88/NF-κB inflammatory pathway, thereby improving cognitive impairment in chronic epilepsy models (Zhou et al., 2025).

Microbiota-related interventions for epilepsy are increasingly being explored within precision medicine frameworks. The ketogenic diet is an established therapy for drug-resistant epilepsy, and emerging evidence suggests that part of its anti-seizure effect may be mediated by changes in the gut microbiome and metabolome (Kowalcze et al., 2025). At the molecular level, ketogenic diet-induced microbial changes have been linked to activation of the SIRT2-PI3K/AKT pathway and neuroprotection (Li et al., 2026b). Microbial signatures may also help predict responses to ketogenic diet treatment in children with drug-resistant epilepsy, supporting the potential value of microbiome-informed patient stratification (Wang Y. et al., 2026). Vagus nerve stimulation and other physical interventions may also interact with gut microbial ecology through bidirectional neural and intestinal pathways. Nevertheless, further human studies are needed to determine whether microbiome changes are causal mediators, biomarkers of treatment response, or secondary effects of dietary and therapeutic interventions.

## 7 Conclusions and perspectives

The microbiota-gut-brain axis provides an integrated framework for understanding how diet, microbial ecology, immune signaling, microbial metabolites, and neural pathways may jointly influence brain function and neuropsychiatric disease vulnerability. Current evidence supports an important association between gut microbial dysbiosis and a range of neurodegenerative, psychiatric, neurodevelopmental, and excitability-related disorders. However, the strength and direction of these associations vary across diseases, study populations, and experimental models. Therefore, microbiota-related findings should be interpreted cautiously, particularly when extrapolating from animal studies to human clinical settings.

Diet appears to be one of the most important modifiable factors shaping the gut microbiota and its functional outputs. Through effects on intestinal barrier integrity, microbial metabolite production, immune homeostasis, and neuroendocrine regulation, dietary patterns may influence MGBA-related pathways relevant to neurological and psychiatric health. Nevertheless, human diet-microbiota-brain interactions are highly heterogeneous and are affected by genetic background, age, medication exposure, lifestyle, geography, disease stage, and baseline microbial composition. These variables remain major challenges for translating mechanistic findings into clinically reliable interventions.

Microbiota-targeted strategies, including precision nutrition, probiotics, prebiotics, fecal microbiota transplantation, metabolite-based approaches, and lifestyle interventions, represent promising but still developing therapeutic directions. At present, many interventions remain supported mainly by preclinical evidence, small clinical studies, or disease-specific observations. Future research should prioritize longitudinal human cohorts, randomized controlled trials, standardized microbiome and metabolomic profiling, clinically meaningful neurological and psychiatric endpoints, and careful patient stratification. Overall, the field is moving toward a more personalized and systems-level understanding of neuropsychiatric disorders, but clinical application will require stronger evidence, mechanistic validation, and rigorous assessment of safety and durability.

## References

[B1] (2017). Correction for Cekanaviciute et al. Gut bacteria from multiple sclerosis patients modulate human T cells and exacerbate symptoms in mouse models. Proc. Natl. Acad. Sci. U.S.A. 114, E8943. doi: 10.1073/pnas.1711235114 29073033 PMC5651795

[B2] Abdelhamid M. Zhou C. Jung C.-G. Michikawa M. (2022). Probiotic Bifidobacterium breve MCC1274 mitigates Alzheimer’s disease-related pathologies in wild-type mice. Nutrients 14, 2543. doi: 10.3390/nu14122543 35745273 PMC9231139

[B3] Aghara H. Patel M. Chadha P. Parwani K. Chaturvedi R. Mandal P. (2025). Unraveling the gut-liver-brain axis: Microbiome, inflammation, and emerging therapeutic approaches. Mediators Inflamm. 2025, 6733477. doi: 10.1155/mi/6733477 40568349 PMC12197523

[B4] AlKuwaiti S. H. Skrabulyte-Barbulescu J. Yassin L. K. Almazrouei S. Aldhaheri D. Alderei M. . (2025). Harnessing the microbiota-gut-brain axis to prevent and treat pediatric neurodevelopmental disorders: translational insights and strategies. J. Transl. Med. 23, 1286. doi: 10.1186/s12967-025-07279-4 41239321 PMC12619268

[B5] Atarashi K. Tanoue T. Oshima K. Suda W. Nagano Y. Nishikawa H. . (2013). Treg induction by a rationally selected mixture of Clostridia strains from the human microbiota. Nature 500, 232–236. doi: 10.1038/nature12331 23842501

[B6] Beam A. Clinger E. Hao L. (2021). Effect of diet and dietary components on the composition of the gut microbiota. Nutrients 13, 2795. doi: 10.3390/nu13082795 34444955 PMC8398149

[B7] Berer K. Mues M. Koutrolos M. Rasbi Z. A. Boziki M. Johner C. . (2011). Commensal microbiota and myelin autoantigen cooperate to trigger autoimmune demyelination. Nature 479, 538–541. doi: 10.1038/nature10554 22031325

[B8] Bertollo A. G. Santos C. F. Bagatini M. D. Ignácio Z. M. (2025). Hypothalamus-pituitary-adrenal and gut-brain axes in biological interaction pathway of the depression. Front. Neurosci. 19, 1541075. doi: 10.3389/fnins.2025.1541075 39981404 PMC11839829

[B9] Biagi E. Candela M. Centanni M. Consolandi C. Rampelli S. Turroni S. . (2014). Gut microbiome in Down syndrome. PloS One 9, e112023. doi: 10.1371/journal.pone.0112023 25386941 PMC4227691

[B10] Borkent J. Ioannou M. Laman J. D. Haarman B. C. M. Sommer I. E. C. (2022). Role of the gut microbiome in three major psychiatric disorders. Psychol. Med. 52, 1222–1242. doi: 10.1017/s0033291722000897 35506416 PMC9157303

[B11] Bruning J. Chapp A. Kaurala G. A. Wang R. Techtmann S. Chen Q.-H. (2020). Gut microbiota and short chain fatty acids: influence on the autonomic nervous system. Neurosci. Bull. 36, 91–95. doi: 10.1007/s12264-019-00410-8 31301036 PMC6940411

[B12] Buffington S. A. Di Prisco G. V. Auchtung T. A. Ajami N. J. Petrosino J. F. Costa-Mattioli M. (2016). Microbial reconstitution reverses maternal diet-induced social and synaptic deficits in offspring. Cell 165, 1762–1775. doi: 10.1016/j.cell.2016.06.001 27315483 PMC5102250

[B13] Cai J. Rimal B. Jiang C. Chiang J. Y. L. Patterson A. D. (2022). Bile acid metabolism and signaling, the microbiota, and metabolic disease. Pharmacol. Ther. 237, 108238. doi: 10.1016/j.pharmthera.2022.108238 35792223

[B14] Chehadi A. C. Pereira de Lima E. Detregiachi C. R. P. Santos de Argollo Haber R. Catharin V. M. C. S. Fornari Laurindo L. . (2026). Harnessing dietary tryptophan: bridging the gap between neurobiology and psychiatry in depression management. Int. J. Mol. Sci. 27, 465. doi: 10.20944/preprints202509.2303.v2 41516336 PMC12787022

[B15] Chen C. Liao J. Xia Y. Liu X. Jones R. Haran J. . (2022). Gut microbiota regulate Alzheimer’s disease pathologies and cognitive disorders via PUFA-associated neuroinflammation. Gut 71, 2233–2252. doi: 10.1136/gutjnl-2021-326269 35017199 PMC10720732

[B16] Chen M. Ruan G. Chen L. Ying S. Li G. Xu F. . (2022). Neurotransmitter and intestinal interactions: focus on the microbiota-gut-brain axis in irritable bowel syndrome. Front. Endocrinol. 13, 817100. doi: 10.3389/fendo.2022.817100 35250873 PMC8888441

[B17] Cignarella F. Cantoni C. Ghezzi L. Salter A. Dorsett Y. Chen L. . (2018). Intermittent fasting confers protection in CNS autoimmunity by altering the gut microbiota. Cell Metab. 27, 1222–1235.e6. doi: 10.1016/j.cmet.2018.05.006 29874567 PMC6460288

[B18] Connell E. Le Gall G. McArthur S. Lang L. Breeze B. Liaquat M. . (2026). A novel Mediterranean diet-inspired supplement reduces hippocampal amyloid deposits and microglial activation through the modulation of the microbiota gut-brain axis in 5xFAD mice. Gut Microbes 18, 2614030. doi: 10.1080/19490976.2026.2614030 41527932 PMC12802989

[B19] Coursan A. Polve D. Leroi A.-M. Monnoye M. Roussin L. Benatar C. . (2026). Fructose malabsorption induces dysbiosis and increases anxiety in male human and animal models. Brain Behav. Immun. 133, 106221. doi: 10.1016/j.bbi.2025.106221 41418890

[B20] Dan Z. Mao X. Liu Q. Guo M. Zhuang Y. Liu Z. . (2020). Altered gut microbial profile is associated with abnormal metabolism activity of Autism Spectrum Disorder. Gut Microbes 11, 1246–1267. doi: 10.1080/19490976.2020.1747329 32312186 PMC7524265

[B21] Dang X. Gong D. Dai S.-S. Teng Z. Luo X.-J. (2026). Genetic and functional insights into long noncoding RNAs in schizophrenia. Mol. Psychiatry 31, 2530–2541. doi: 10.1038/s41380-025-03421-2 41392096

[B22] Duscha A. Gisevius B. Hirschberg S. Yissachar N. Stangl G. I. Dawin E. . (2020). Propionic acid shapes the multiple sclerosis disease course by an immunomodulatory mechanism. Cell 180, 1067–1080.e16. doi: 10.1016/j.cell.2020.02.035 32160527

[B23] Dziedziak M. Mytych A. Szyller H. P. Lasocka M. Augustynowicz G. Szydziak J. . (2025). Gut microbiota in psychiatric and neurological disorders: Current insights and therapeutic implications. Biomedicines 13, 2104. doi: 10.3390/biomedicines13092104 41007667 PMC12467032

[B24] Fitzpatrick Z. Frazer G. Ferro A. Clare S. Bouladoux N. Ferdinand J. . (2020). Gut-educated IgA plasma cells defend the meningeal venous sinuses. Nature 587, 472–476. doi: 10.1038/s41586-020-2886-4 33149302 PMC7748383

[B25] Fond G. B. Lagier J.-C. Honore S. Lancon C. Korchia T. Sunhary De Verville P.-L. . (2020). Microbiota-orientated treatments for major depression and schizophrenia. Nutrients 12, 1024. doi: 10.3390/nu12041024 32276499 PMC7230529

[B26] Fu R. Liang X.-J. Yang W.-M. Li R. Shi Y.-R. Guo L. . (2026). Gut microbial signatures in schizophrenia: Exploring archaea, fungi, and bacteria. BMC Psychiatry 26, 113. doi: 10.1186/s12888-025-07721-3 41484966 PMC12866035

[B27] He N. Zhong J. Deng S. Liang J. Li Q. Chen K. (2025). The impact of trans fatty acids on ADHD in relation to the gut microbiome. Front. Nutr. 12, 1641574. doi: 10.3389/fnut.2025.1641574 41346685 PMC12673567

[B28] Hong S. Yu M. Zheng Y. Wang S. Zhang J. Pan B. . (2026). Clinical efficacy and multi-omics analysis of Si-Ni-San for depression treatment in breast cancer patients: A randomized, double-blind, placebo-controlled, crossover trial. Chin. Med. 21, 9. doi: 10.1186/s13020-025-01283-y 41491244 PMC12771921

[B29] Horsager J. Andersen K. B. Knudsen K. Skjærbæk C. Fedorova T. D. Okkels N. . (2020). Brain-first versus body-first Parkinson’s disease: a multimodal imaging case-control study. Brain J. Neurol. 143, 3077–3088. doi: 10.1093/brain/awaa238 32830221

[B30] Hou Y. Li J. Ying S. (2023). Tryptophan metabolism and gut microbiota: a novel regulatory axis integrating the microbiome, immunity, and cancer. Metabolites 13, 1166. doi: 10.3390/metabo13111166 37999261 PMC10673612

[B31] Hsiao E. Y. McBride S. W. Hsien S. Sharon G. Hyde E. R. McCue T. . (2013). Microbiota modulate behavioral and physiological abnormalities associated with neurodevelopmental disorders. Cell 155, 1451–1463. doi: 10.1016/j.cell.2013.11.024 24315484 PMC3897394

[B32] Huang L. Zheng Y. Liu Q. Feng Y. Ma Z. Zhao X. . (2026a). Milk fat globule membrane ameliorates depressive-like behaviors in chronic unpredictable mild stress rats by modulating the microbiota-gut-brain axis. Biosci. Microbiota Food. Health 45, 66–78. doi: 10.12938/bmfh.2025-031 41492376 PMC12765539

[B33] Huang Z. Huang Z. Du Z. Gao X. Jiang Y. Zhou Z. . (2026b). Role and mechanism of gut microbiota and metabolites in schizophrenia complicated with sleep disorder. Gut Microbes 18, 2607817. doi: 10.1080/19490976.2025.2607817 41459834 PMC12758268

[B34] Huynh V. A. Takala T. M. Murros K. E. Diwedi B. Saris P. E. J. (2023). Desulfovibrio bacteria enhance alpha-synuclein aggregation in a Caenorhabditis elegans model of Parkinson’s disease. Front. Cell. Infect. Microbiol. 13, 1181315. doi: 10.3389/fcimb.2023.1181315 37197200 PMC10183572

[B35] iMSMS Consortium (2022). Gut microbiome of multiple sclerosis patients and paired household healthy controls reveal associations with disease risk and course. Cell 185, 3467–3486.e16. doi: 10.1016/j.cell.2022.08.021 36113426 PMC10143502

[B36] Ivanov I. I. Atarashi K. Manel N. Brodie E. L. Shima T. Karaoz U. . (2009). Induction of intestinal Th17 cells by segmented filamentous bacteria. Cell 139, 485–498. doi: 10.1016/j.cell.2009.09.033 19836068 PMC2796826

[B37] Jangi S. Gandhi R. Cox L. M. Li N. von Glehn F. Yan R. . (2016). Alterations of the human gut microbiome in multiple sclerosis. Nat. Commun. 7, 12015. doi: 10.1038/ncomms12015 27352007 PMC4931233

[B38] Jia H. Guo X. Wei Y. Can C. He N. Zhang H. . (2025). Chronic stress, gut microbiota, and immunity: interconnections and implications for health. Mol. Cell. Biochem. 480, 5995–6014. doi: 10.1007/s11010-025-05376-y 40864384

[B39] Ju T. Zhang Y. Liu L. Zhao X. Li X. Liu C. . (2026). The role of gut microbiota-mitochondria crosstalk in neurodegeneration: underlying mechanisms and potential therapies. Neural Regener. Res. 21, 2238–2253. doi: 10.4103/nrr.nrr-d-24-01419 40314217 PMC13211786

[B40] Kanaya A. Luković E. Emala C. Mikami M. (2025). Effects of chronic allergic lung inflammation on gut microbiota and depression-like behavior in mice. Explor. Asthma Allergy 3, 100978. doi: 10.37349/eaa.2025.100978 41523930 PMC12781662

[B41] Kang D.-W. Adams J. B. Coleman D. M. Pollard E. L. Maldonado J. McDonough-Means S. . (2019). Long-term benefit of microbiota transfer therapy on autism symptoms and gut microbiota. Sci. Rep. 9, 5821. doi: 10.1038/s41598-019-42183-0 30967657 PMC6456593

[B42] Kang J. W. Vemuganti V. Kuehn J. F. Ulland T. K. Rey F. E. Bendlin B. B. (2024). Gut microbial metabolism in Alzheimer’s disease and related dementias. Neurother. J. Am. Soc Exp. Neurother. 21, e00470. doi: 10.1016/j.neurot.2024.e00470 39462700 PMC11585892

[B43] Kim S. Kim H. Yim Y. S. Ha S. Atarashi K. Tan T. G. . (2017). Maternal gut bacteria promote neurodevelopmental abnormalities in mouse offspring. Nature 549, 528–532. doi: 10.1038/nature23910 28902840 PMC5870873

[B44] Kim S. Kwon S.-H. Kam T.-I. Panicker N. Karuppagounder S. S. Lee S. . (2019). Transneuronal propagation of pathologic α-synuclein from the gut to the brain models Parkinson’s disease. Neuron 103, 627–641.e7. doi: 10.1016/j.neuron.2019.05.035 31255487 PMC6706297

[B45] Koh A. De Vadder F. Kovatcheva-Datchary P. Bäckhed F. (2016). From dietary fiber to host physiology: short-chain fatty acids as key bacterial metabolites. Cell 165, 1332–1345. doi: 10.1016/j.cell.2016.05.041 27259147

[B46] Korenblik V. Schilder N. K. M. de Lange I. G. S. Daams J. G. Bockting C. L. H. Brul S. . (2026). From gut to glee: Is butyrate a promising antidepressant? A systematic review and mechanistic insights. Brain Behav. Immun. 132, 106237. doi: 10.1016/j.bbi.2025.106237 41429215

[B47] Kowalcze K. Dyńka D. Klus W. Dudzińska M. Paziewska A. (2025). Modulation of gut microbiome and metabolome as one of the potential mechanisms of ketogenic diet effect in the treatment of epilepsy. Nutrients 18, 31. doi: 10.3390/nu18010031 41515148 PMC12787722

[B48] Kuai X.-Y. Yao X.-H. Xu L.-J. Zhou Y.-Q. Zhang L.-P. Liu Y. . (2021). Evaluation of fecal microbiota transplantation in Parkinson’s disease patients with constipation. Microb. Cell. Factories 20, 98. doi: 10.1186/s12934-021-01589-0 33985520 PMC8120701

[B49] Lan J. Wang J. Huang S. Li C. Deng Z. Hao Z. . (2026). Neuropeptide SP protects against colitis and linked anxiety-like behavior through the putative roles of gut microbiota and metabolite inositol. Nat. Commun. 17, 295. doi: 10.1038/s41467-025-67904-0 41507168 PMC12789692

[B50] Lanzara R. Conti C. Zito L. Anaclerio F. Affaitati G. P. Giamberardino M. A. . (2026). Gut microbiota and psychological distress in fibromyalgia: A systematic review. Biopsychosoc Sci. Med. 88, 287–296. doi: 10.1097/psy.0000000000001463 41504323

[B51] Lawrence K. Fibert P. Toribio-Mateas M. Gregory A. M. Hobbs J. Quadt F. . (2025). Effects of kefir on symptoms, sleep, and gut microbiota in children with ADHD: a randomised controlled trial. BMC Psychiatry 25, 1117. doi: 10.1186/s12888-025-07568-8 41286799 PMC12642372

[B52] Lee M.-C. Chen C.-Y. Chen C.-Y. Huang C.-C. (2025). Heat-treated Limosilactobacillus fermentum PS150 improves sleep quality with severity-dependent benefits: A randomized, placebo-controlled trial. Nutrients 18, 14. doi: 10.3390/nu18010014 41515133 PMC12787598

[B53] Li H. Niu R. He W. Lai H. Zou S. Zou Q. . (2026a). Lachnospira eligens attenuates epileptogenesis via gut-brain axis regulation of blood-brain barrier integrity and neuroinflammation. Theranostics 16, 1045–1062. doi: 10.7150/thno.116959 41356796 PMC12675144

[B54] Li M. Deng H. Ma L. Sun M. (2026b). The anti-epileptic mechanism of a ketogenic diet regulating the gut microbiota via SIRT2 activation of the PI3K/AKT signaling pathway. Neurosci. Lett. 872, 138488. doi: 10.1016/j.neulet.2025.138488 41380905

[B55] Li X. Ji J. Li J. Li S. Luo Q. Gu M. . (2025a). Gut microbiota-bile acid metabolic disorder involved in the cognitive impairments in epilepsy through HO-1 dependent ferroptosis. J. Pharm. Anal. 15, 101291. doi: 10.1016/j.jpha.2025.101291 41399411 PMC12702009

[B56] Li Y. Wu H. Jia H. Li C. Song S. Jia S. . (2025b). Effects of dietary patterns and enterotypes on frailty-related metabolic indicators in elderly diabetics. Wei Sheng Yan Jiu 54, 923–944. doi: 10.19813/j.cnki.weishengyanjiu.2025.06.007 41506985

[B57] Li Z. Qin P. Sun Z. Li L. Liang P. Zhao Y. . (2026c). Distinct effects of different Bacteroides strains on depressive-like behavior via a gut-Th1/Th17 cells-brain axis. Commun. Biol. 9, 247. doi: 10.1038/s42003-026-09525-x 41530303 PMC12905409

[B58] Marano G. Traversi G. Mazza O. Caroppo E. Capristo E. Gaetani E. . (2025). The immune mind: Linking dietary patterns, microbiota, and psychological health. Nutrients 18, 96. doi: 10.3390/nu18010096 41515213 PMC12787503

[B59] Mendóza R. Santos J. M. Liu X. Elmassry M. M. Ji G. Kiritoshi T. . (2026). Gingerol-enriched ginger extract effects on anxiety-like behavior in a neuropathic pain model via colonic microbiome-neuroimmune modulation. Molecules 31, 166. doi: 10.3390/molecules31010166 41515464 PMC12787805

[B60] Minami T. Ikegame T. Tanaka M. Kumagai E. Kanehara A. Morishima R. . (2025). Urinary metabolomic profiling in 22q11.2 deletion syndrome reveals microbial and mitochondrial signatures related to autism and psychosis risk. PCN Rep. Psychiatry Clin. Neurosci. 4, e70261. doi: 10.1002/pcn5.70261 41346956 PMC12673154

[B61] Miyake S. Kim S. Suda W. Oshima K. Nakamura M. Matsuoka T. . (2015). Dysbiosis in the gut microbiota of patients with multiple sclerosis, with a striking depletion of species belonging to Clostridia XIVa and IV clusters. PloS One 10, e0137429. doi: 10.1371/journal.pone.0137429 26367776 PMC4569432

[B62] Morton J. T. Jin D.-M. Mills R. H. Shao Y. Rahman G. McDonald D. . (2023). Multi-level analysis of the gut-brain axis shows autism spectrum disorder-associated molecular and microbial profiles. Nat. Neurosci. 26, 1208–1217. doi: 10.1016/s0016-0032(69)90237-3 37365313 PMC10322709

[B63] Nayak A. Bera S. Purohit S. Jain C. K. (2026). Gut microbiota mediated neuroinflammation in psychiatric disorders: Current perspectives and challenges. Behav. Brain Res. 501, 116019. doi: 10.1016/j.bbr.2025.116019 41475542

[B64] Needham B. D. Funabashi M. Adame M. D. Wang Z. Boktor J. C. Haney J. . (2022). A gut-derived metabolite alters brain activity and anxiety behaviour in mice. Nature 602, 647–653. doi: 10.1038/s41586-022-04396-8 35165440 PMC9170029

[B65] Nguyen T. T. Baumann P. Tüscher O. Schick S. Endres K. (2023). The aging enteric nervous system. Int. J. Mol. Sci. 24, 9471. doi: 10.3390/ijms24119471 37298421 PMC10253713

[B66] Ning B. Yang L. Wei Y. Luo C. Zheng F. Ge T. . (2025). Clinical efficacy and mechanisms of transcutaneous auricular vagus nerve stimulation targeting the gut-brain axis for postoperative complications of aortic dissection: Study protocol for a randomized controlled trial. Front. Med. 12, 1692356. doi: 10.3389/fmed.2025.1692356 41488091 PMC12756893

[B67] Ottaviano E. Marsiglia M. D. Ceccarani C. Ancona S. Triva F. La Briola F. . (2025). Gut microbiota signatures in tuberous sclerosis complex and epilepsy: a pilot study. Front. Neurosci. 19, 1655456. doi: 10.3389/fnins.2025.1655456 41341265 PMC12670250

[B68] Pan D. Jiang M. Wang Y. He J. Tang J. Liu S. . (2026). Multi-omics reveals associations between the microbiota-gut-brain axis and antidepressant effects of vagus nerve stimulation. Neurobiol. Stress 40, 100777. doi: 10.1016/j.ynstr.2025.100777 41492358 PMC12765250

[B69] Patrono E. Svoboda J. Stuchlík A. (2021). Schizophrenia, the gut microbiota, and new opportunities from optogenetic manipulations of the gut-brain axis. Behav. Brain Funct. BBF 17, 7. doi: 10.1186/s12993-021-00180-2 34158061 PMC8218443

[B70] Planas R. Santos R. Tomas-Ojer P. Cruciani C. Lutterotti A. Faigle W. . (2018). GDP-l-fucose synthase is a CD4+ T cell-specific autoantigen in DRB3*02:02 patients with multiple sclerosis. Sci. Transl. Med. 10, eaat4301. doi: 10.1126/scitranslmed.aat4301 30305453

[B71] Qi X. Yun C. Pang Y. Qiao J. (2021). The impact of the gut microbiota on the reproductive and metabolic endocrine system. Gut Microbes 13, 1–21. doi: 10.1080/19490976.2021.1894070 PMC7971312 33722164

[B72] Qiao Y. Liang X. Cheng R. Zhang X. Guo J. Wang Q. . (2026). Time-dependent multisystem effects of small independent space (SIS) exposure in mice: Integrative analysis of behavior, neuroendocrine, gut microbiota, and hippocampal function. FASEB J. Off. Publ. Fed. Am. Soc Exp. Biol. 40, e71411. doi: 10.1096/fj.202503400rr 41527764

[B73] Qin Q. Teng Z. Liu C. Li Q. Yin Y. Tang Y. (2021). TREM2, microglia, and Alzheimer’s disease. Mech. Ageing Dev. 195, 111438. doi: 10.1016/j.mad.2021.111438 33516818

[B74] Ren S. Wang X. Qin J. Mu Q. Ye S. Zhang Y. . (2022). Altered gut microbiota correlates with cognitive impairment in Chinese children with Down’s syndrome. Eur. Child Adolesc. Psychiatry 31, 189–202. doi: 10.1007/s00787-021-01799-2 33999314 PMC8816804

[B75] Robert M. Saha S. Dizman N. Rohlfs M. Sirmans E. Simon J. . (2025). Investigating chronic toxicity, diet, patient-reported outcomes and the microbiome in immunotherapy-treated metastatic melanoma survivors: A new frontier. Nutrients 18, 40. doi: 10.3390/nu18010040 41515159 PMC12787744

[B76] Ronan V. Yeasin R. Claud E. C. (2021). Childhood development and the microbiome-the intestinal microbiota in maintenance of health and development of disease during childhood development. Gastroenterology 160, 495–506. doi: 10.1053/j.gastro.2020.08.065 33307032 PMC8714606

[B77] Rosso R. Virgilio E. Bronzini M. Rolla S. Maglione A. Clerico M. (2026). The hidden players in multiple sclerosis nutrition: a narrative review on the influence of vitamins, polyphenols, salt, and essential metals on disease and gut microbiota. Nutrients 18, 148. doi: 10.3390/nu18010148 41515264 PMC12788138

[B78] Salamon A. Szabó A. Felföldi T. Bel Rhali S. Andics A. Miklósi Á. . (2025). Human-like associations between gut microbiome composition and inattention, hyperactivity, and impulsivity in dogs. BMC Biol. 23, 352. doi: 10.1186/s12915-025-02410-9 41299418 PMC12661682

[B79] Sampson T. R. Debelius J. W. Thron T. Janssen S. Shastri G. G. Ilhan Z. E. . (2016). Gut microbiota regulate motor deficits and neuroinflammation in a model of Parkinson’s disease. Cell 167, 1469–1480.e12. doi: 10.1016/j.cell.2016.11.018 27912057 PMC5718049

[B80] Sanmarco L. M. Wheeler M. A. Gutiérrez-Vázquez C. Polonio C. M. Linnerbauer M. Pinho-Ribeiro F. A. . (2021). Gut-licensed IFNγ+ NK cells drive LAMP1+TRAIL+ anti-inflammatory astrocytes. Nature 590, 473–479. doi: 10.1038/s41586-020-03116-4 33408417 PMC8039910

[B81] Seo D.-O. O’Donnell D. Jain N. Ulrich J. D. Herz J. Li Y. . (2023). ApoE isoform- and microbiota-dependent progression of neurodegeneration in a mouse model of tauopathy. Science 379, eadd1236. doi: 10.1126/science.add1236 36634180 PMC9901565

[B82] Sgritta M. Dooling S. W. Buffington S. A. Momin E. N. Francis M. B. Britton R. A. . (2019). Mechanisms underlying microbial-mediated changes in social behavior in mouse models of autism spectrum disorder. Neuron 101, 246–259.e6. doi: 10.1016/j.neuron.2018.11.018 30522820 PMC6645363

[B83] Sharon G. Cruz N. J. Kang D.-W. Gandal M. J. Wang B. Kim Y.-M. . (2019). Human gut microbiota from autism spectrum disorder promote behavioral symptoms in mice. Cell 177, 1600–1618.e17. doi: 10.1016/j.cell.2019.05.004 31150625 PMC6993574

[B84] Tan A. H. Hor J. W. Chong C. W. Lim S.-Y. (2021). Probiotics for Parkinson’s disease: Current evidence and future directions. JGH Open Open Access J. Gastroenterol. Hepatol. 5, 414–419. doi: 10.1002/jgh3.12450 PMC8035463 33860090

[B85] Tang T. W. H. Ullah K. Lee J.-J. Chen H.-C. Hsieh P. C. H. (2026). Comparative insights into the gut-heart axis: cross-species and cross-population perspectives. Gut Microbes 18, 2611617. doi: 10.1080/19490976.2025.2611617 41520281 PMC12795268

[B86] Tofani G. S. S. Leigh S.-J. Gheorghe C. E. Bastiaanssen T. F. S. Wilmes L. Sen P. . (2025). Gut microbiota regulates stress responsivity via the circadian system. Cell Metab. 37, 138–153.e5. doi: 10.1016/j.cmet.2024.10.003 39504963

[B87] Uğur K. (2025). The influence of endocrine disruptors on the gut microbiota. Turk. J. Med. Sci. 55, 1635–1640. doi: 10.55730/1300-0144.6124 41488241 PMC12758922

[B88] Vara-Pérez M. Movahedi K. (2025). Border-associated macrophages as gatekeepers of brain homeostasis and immunity. Immunity 58, 1085–1100. doi: 10.1016/j.immuni.2025.04.005 40324381 PMC12094687

[B89] Vuong H. E. Pronovost G. N. Williams D. W. Coley E. J. L. Siegler E. L. Qiu A. . (2020). The maternal microbiome modulates fetal neurodevelopment in mice. Nature 586, 281–286. doi: 10.1038/s41586-020-2745-3 32968276 PMC7554197

[B90] Wang R. Hou L. Wu Q. Wen X. Xiong Y. Yang X. . (2025a). The role of CP level and interaction with antibiotics in the post-weaning piglets’ diet: growth performance, body composition, nutrient digestion, and intestinal health. Anim. Open Access J. MDPI 16, 24. doi: 10.3390/ani16010024 PMC12784924 41514712

[B91] Wang X. Liu M. Cao L. Huang H. Yang J. Zhang H. . (2025b). Gut microbiota-neuroinflammation axis: a new mechanism and therapeutic target for comorbid depression in epilepsy. Brain Behav. Immun. - Health 50, 101150. doi: 10.1016/j.bbih.2025.101150 41476669 PMC12750504

[B92] Wang X. Wu Q. Zeng H.-L. Shen Y. Zhang H.-H. Gong J. . (2026a). Gut bacteria improve depressive symptoms by degrading cortisol into androgen. Adv. Sci. 13, e08468. doi: 10.1002/advs.202508468 41504155 PMC12931197

[B93] Wang Y. Guo J. Zhang P. Zhou Q. Peng K. Xu Z. . (2026b). Gut microbiota signatures associated with ketogenic diet response in pediatric drug-resistant epilepsy. Seizure 134, 220–228. doi: 10.1016/j.seizure.2025.12.012 41468651

[B94] Wang Y.-H. Kong X.-R. Ye X.-Z. Pu S. Ren X.-X. Huang Q.-Y. . (2025c). Exploring the animal models of gastrointestinal and emotional comorbidity. Brain Behav. 15, e71135. doi: 10.1002/brb3.71135 41405490 PMC12710529

[B95] Wastyk H. C. Fragiadakis G. K. Perelman D. Dahan D. Merrill B. D. Yu F. B. . (2021). Gut-microbiota-targeted diets modulate human immune status. Cell 184, 4137–4153.e14. doi: 10.1016/j.cell.2021.06.019 34256014 PMC9020749

[B96] Wu X. Chen W. Yong Q. Zeng Q. You J. Zeng K. . (2026). Limosilactobacillus reuteri NCU-37 alleviates leuprorelin-induced perimenopausal syndrome in infertile women by modulating the gut microbiota: a randomized controlled trial. Food Funct. 17, 565–576. doi: 10.1039/d5fo04330a 41399984

[B97] Wu Y. Zang S. Wu Z. Huang J. (2025). First exposure to second-generation antipsychotics alters gut microbiota and metabolic profiles in patients with glucose-lipid metabolism disorders. Front. Psychiatry 16, 1722760. doi: 10.3389/fpsyt.2025.1722760 41446296 PMC12723006

[B98] Xiao Y. Wei L. Yu J. Liu Y. (2025). Fecal microbiota transplantation for attention-deficit/hyperactivity disorder: mechanisms, evidence, and future directions. Int. J. Gen. Med. 18, 6757–6767. doi: 10.2147/ijgm.s548322 41229548 PMC12604505

[B99] Xiao L. Yan J. Yang T. Zhu J. Li T. Wei H. . (2021). Fecal microbiome transplantation from children with autism spectrum disorder modulates tryptophan and serotonergic synapse metabolism and induces altered behaviors in germ-free mice. mSystems 6, e01343-20. doi: 10.1128/msystems.01343-20 33824200 PMC8547010

[B100] Xie Y.-A. Kong J.-D. Li S. Wei D.-F. (2025). What is the impact of dopamine D2 receptor in the brain-gut axis? A narrative review of the mechanism based on gut microbiota in modulating emotion and behavior. Alpha Psychiatry 26, 39226. doi: 10.31083/ap39226 41523964 PMC12781235

[B101] Xu C. Kong L. Mou T. Tang A. Hu S. Lai J. (2025a). Vitamin B12 and affective disorders: A focus on the gut-brain axis. Alpha Psychiatry 26, 49138. doi: 10.31083/AP49138 41523970 PMC12781227

[B102] Xu K. Ren Y. Zhao S. Ren Z. Wang J. Tu D. . (2025b). Consistent decline of acetylcholine in microbiota-gut-brain axis mediates antibiotic-induced anxiety via regulating hippocampus microglial activation. Mol. Psychiatry 31, 2965–2977. doi: 10.1038/s41380-025-03431-0 41436582

[B103] Yang C. Bao L. Shi Z. Xv X. Li J. Jiang D. . (2026). Jingning formula alleviates ADHD by restoring gut microbiota dysbiosis and tryptophan metabolic dysfunction. J. Pharm. Biomed. Anal. 269, 117256. doi: 10.1016/j.jpba.2025.117256 41264968

[B104] Yap C. X. Henders A. K. Alvares G. A. Wood D. L. A. Krause L. Tyson G. W. . (2021). Autism-related dietary preferences mediate autism-gut microbiome associations. Cell 184, 5916–5931.e17. doi: 10.52843/cassyni.15kd14 34767757

[B105] Yuan Y.-L. Lan Y.-M. Guo L.-M. (2025). The microbiota-gut-brain axis in childhood attention-deficit/hyperactivity disorder: mechanisms and therapeutic advances. Zhongguo Dang Dai Er Ke Za Zhi Chin. J. Contemp. Pediatr. 27, 1426–1432. doi: 10.7499/j.issn.1008-8830.2503118 PMC12688203 41250545

[B106] Zang J. Xiao L. Shi Y. Kou Y. Ma K. Zhang C. . (2026). Advances in dietary modulation of the intestinal barrier: mechanistic, structural, and functional insights. Compr. Rev. Food Sci. Food Saf. 25, e70383. doi: 10.1111/1541-4337.70383 41511109

[B107] Zeng S.-Y. Liu J.-H. Xiang Y.-Y. Zeng Z.-L. Zhao Z.-B. Zheng J. . (2026). A novel protein B2URF3 from Akkermansia muciniphila increased by intermittent fasting alleviates vascular calcification. J. Nanobiotechnology 24, 21. doi: 10.1186/s12951-025-03948-0 41501766 PMC12781770

[B108] Zha X. Liu X. Wei M. Huang H. Cao J. Liu S. . (2025). Microbiota-derived lysophosphatidylcholine alleviates Alzheimer’s disease pathology via suppressing ferroptosis. Cell Metab. 37, 169–186.e9. doi: 10.1016/j.cmet.2024.10.006 39510074

[B109] Zhang T. Gao G. Kwok L.-Y. Sun Z. (2023). Gut microbiome-targeted therapies for Alzheimer’s disease. Gut Microbes 15, 2271613. doi: 10.1080/19490976.2023.2271613 37934614 PMC10631445

[B110] Zhang Z. Hu X. Tao W. Ma R. Zheng Y. Fang X. . (2026). Host-gut microbial metabolic crosstalk in postpartum depression: A multiomics insight linking blood metabolites to epigenetic modulation. J. Affect. Disord. 400, 121166. doi: 10.1016/j.jad.2026.121166 41519171

[B111] Zhao J. Liu J. Feng J. Liu X. Hu Q. (2024). The gut microbiota-brain connection: Insights into major depressive disorder and bipolar disorder. Front. Psychiatry 15, 1421490. doi: 10.3389/fpsyt.2024.1421490 39564459 PMC11574523

[B112] Zheng Y. Qu Y. Yao M. Li K. Dong Y. Xing X. . (2025). Mechanisms of aerobic exercise effects on the gut microbiota and its metabolites in anxiety disorders. Front. Microbiol. 16, 1721497. doi: 10.3389/fmicb.2025.1721497 41459223 PMC12740242

[B113] Zhou S. Guo L. Chen N. Liu H. Liu X. Li J. . (2026). Depression aggravates immune-mediated hepatitis through NLRP3 overactivation induced by intestinal microbiota. CNS Neurosci. Ther. 32, e70743. doi: 10.1002/cns.70743 41503678 PMC12780859

[B114] Zhou K. Wang Y. Sun W. Mao Z. Wang W. Qu Z. (2025). Metformin alleviates cognitive impairment in chronic-phase epileptic rats by modulating the TLR4/MyD88/NF-κB pathway and gut flora composition. Mol. Neurobiol. 63, 272. doi: 10.1007/s12035-025-05516-x 41359249

[B115] Zierfuss B. Larochelle C. Prat A. (2024). Blood-brain barrier dysfunction in multiple sclerosis: causes, consequences, and potential effects of therapies. Lancet Neurol. 23, 95–109. doi: 10.1016/S1474-4422(23)00377-0 38101906

